# Elevated Kallistatin Induces Myosteatosis and Exercise Intolerance by Antagonizing AdipoR1‐Mediated AMPK Signalling

**DOI:** 10.1002/jcsm.70261

**Published:** 2026-04-01

**Authors:** Fuyan Hong, Zhenzhen Fang, Gang Shen, Yan Wang, Yunhua Li, Youbin Zhong, Junze Dai, Chengwei Zhang, Jing Zhang, Wencan Chen, Weiwei Qi, Xia Yang, Guoquan Gao, Ti Zhou

**Affiliations:** ^1^ Department of Biochemistry and Molecular Biology, Zhongshan School of Medicine Sun Yat‐sen University Guangzhou China; ^2^ Department of Laboratory Medicine The Second Affiliated Hospital of Guangzhou University of Chinese Medicine Guangzhou China; ^3^ Department of Laboratory Medicine Third Affiliated Hospital of Sun Yat‐Sen University Guangzhou China; ^4^ Guangdong Engineering and Technology Research Center for Gene Manipulation and Bio‐macromolecular Products Sun Yat‐Sen University Guangzhou China; ^5^ China Key Laboratory of Tropical Disease Control (Sun Yat‐sen University), Ministry of Education Guangzhou China; ^6^ Guangdong Provincial Key Laboratory of Diabetology Guangzhou Guangdong China

**Keywords:** AdipoR1, AMPK, kallistatin, lipogenesis, mitochondrial biogenesis, myosteatosis

## Abstract

**Background:**

Pathological intramuscular lipid deposition (myosteatosis) and exercise intolerance are hallmarks of metabolic disorders, including diabetes and metabolic dysfunction‐associated steatotic liver disease (MASLD), yet their underlying mechanisms remain unclear. Our previous work has confirmed that hypertriglyceridemia‐driven kallistatin (KAL) elevation is present in the peripheral blood of patients with MASLD and diabetes and is a causative factor in hepatic steatosis and MASH pathogenesis. Here, we aim to evaluate this elevated KAL on myosteatosis and exercise function.

**Methods:**

We first established rat models of myosteatosis via a high‐fat diet or high‐fructose water intake and measured serum KAL levels by immunoblotting. KAL transgenic (KAL‐TG) mice were generated and subjected to longitudinal analyses, including Oil Red O staining for lipid deposition, exercise tolerance tests, indirect calorimetry for energy expenditure, mitochondrial DNA copy number quantification, ATP measurement, immunoblotting and quantitative PCR. For mechanistic analyses, mouse C2C12 myotubes were treated with recombinant KAL or KAL adenovirus, with or without co‐treatment with AICAR, AdipoRon or AdipoR1 siRNA. Additionally, we evaluated the potential ameliorative effects of KAL knockout and target agonists on myosteatosis.

**Results:**

Serum KAL levels were significantly elevated in rat models of myosteatosis. Conversely, genetic ablation of KAL ameliorated diet‐induced muscle lipid deposition. KAL‐TG mice developed myosteatosis (muscle TG [μmol/g]: WT: 54.26 ± 14.56 [95% CI: 38.99–69.54]; KAL‐TG: 85.13 ± 11.53 [95% CI: 73.03–97.24], *p* < 0.01) and exhibited exercise intolerance (*p* < 0.05 for all) from 6 months of age. Mechanistically, KAL bound to sarcolemmal adiponectin receptor 1 (AdipoR1), suppressing AMPK activity. This led to reduced phosphorylation of acetyl‐CoA carboxylase (ACC) (*p* < 0.05), enhancing lipogenesis and downregulated the PGC‐1α/NRF1 axis (*p* < 0.05), impairing mitochondrial biogenesis and reducing ATP production (*p* < 0.05). Pharmacological activation of AdipoR1 (AdipoRon) and fenofibrate attenuated myosteatosis and restored exercise capacity in KAL‐TG mice (*p* < 0.05).

**Conclusions:**

Abnormal elevation of KAL drives metabolic myopathy and exercise intolerance by antagonizing the AdipoR1‐AMPK axis. Our findings offer dual strategies: repurposing AdipoR1 agonists (e.g., AdipoRon) or reducing circulating KAL (e.g., via genetic ablation or triglyceride‐lowering agents such as fenofibrate), both applicable to diabetes/MASLD‐related metabolic myopathy.

## 1 Introduction

Type 2 diabetes mellitus (T2DM), hypertriglyceridemia (HTG) and metabolic dysfunction‐associated steatotic liver disease (MASLD) represent a growing triad of interconnected metabolic disorders that pose significant public health challenges worldwide. These conditions frequently co‐occur, creating a synergistic effect that accelerates disease progression. Current epidemiological data indicate that both HTG and MASLD affect approximately 30% of the global adult population, with prevalence rates continuing to rise [1, 2]. The global burden of diabetes is equally concerning, with recent estimates projecting 828 million affected adults in 2022, corresponding to a prevalence rate of 13.9% [3]. A common pathological feature of these metabolic disorders is intramuscular lipid deposition, which is accompanied by reduced muscle strength [4, 5].

Myosteatosis, characterized by pathological fat accumulation within skeletal muscle tissue, leading to impaired metabolic function and compromised musculoskeletal health, has emerged as a distinct clinical entity separate from sarcopenia. Emerging evidence suggests a high prevalence of muscle lipid deposition in metabolic disorders, with 21.1% of MASLD patients exhibiting severe intramuscular fat accumulation. Notably, the prevalence increases to 33.3% in patients with early‐stage metabolic dysfunction‐associated steatohepatitis (MASH), indicating a potential progression‐related pattern [6]. While comprehensive population‐based epidemiological data on myosteatosis remain limited, its clinical significance is increasingly recognized, given its strong associations with HTG, MASLD and T2DM.

Myosteatosis profoundly impacts clinical outcomes. Intramuscular fat accumulation directly impairs muscle strength through structural changes and molecular dysregulation, including the PKC‐mediated suppression of IRS‐1/PI3K insulin signalling [7, 8]. This initiates a vicious cycle, exacerbating hyperglycemia and hyperlipidemia (HLP) [9]. Critically, myosteatosis independently predicts disease progression and all‐cause mortality in MASLD/diabetes even in asymptomatic individuals [6, 10, 11, 12, 13]. Reduced muscle strength further compromises the effectiveness of exercise‐based interventions, highlighting the urgent need for therapies that target both metabolic and functional consequences in the management of HTG, MASLD and T2DM.

The molecular mechanisms driving myosteatosis remain incompletely characterized. While fatty acid uptake, mitochondrial dysfunction and fibro‐adipogenic progenitor differentiation contribute to intramuscular lipid accumulation [14, 15], the role of de novo lipogenesis is poorly defined. Our previous study identified markedly elevated circulating kallistatin (KAL) levels in HTG patients—a finding replicated in MASLD cohorts [16]. We subsequently demonstrated that KAL promotes hepatic steatosis by suppressing lipolysis. Notably, diabetic patients (who exhibit high myosteatosis prevalence) show similarly elevated serum KAL levels correlating with impaired wound healing [17]. As a hepatically derived SERPIN family member, KAL expression is upregulated by free fatty acid (FFA) accumulation [16]. However, whether KAL contributes to myosteatosis and exercise intolerance in these metabolic disorders remains unexplored.

In this study, we investigated the mechanistic role of KAL in muscle lipid metabolism and exercise function, revealing novel therapeutic strategies for diabetes‐ and MASLD‐associated myosteatosis.

## 2 Methods

### 2.1 Animals

All animal procedures were approved by the Sun Yat‐sen University IACUC (SYSU‐IACUC‐2022‐B0016). C57BL/6 wild‐type (WT) mice were obtained from Guangdong Experimental Animal Center, while KAL transgenic (KAL‐TG) mice were generously provided by Dr. Jianxing Ma (University of Oklahoma) [18]. Briefly, this transgenic mouse line expressing human KAL ubiquitously was generated using the β‐actin promoter and was identified by PCR genotyping with the following transgene‐specific primers: forward 5′‐AGGGAAGATTGGATTTGG‐3′ and reverse 5′‐AGGAGAGATCAGTGATGCTC‐3′. KAL knockout rats (*Serpina4* ^−^/^−^) were generated via CRISPR/Cas9 by Guangzhou Saiye Biotechnology. Briefly, a pair of sgRNAs was designed to direct the Cas9 nuclease to create double‐strand breaks within exons 2 and 3 of the rat *Serpina4* gene (which encodes the KAL protein). The subsequent cellular repair via nonhomologous end joining (NHEJ) resulted in the complete deletion of the intervening genomic sequence between these two target sites, effectively removing the entirety of exons 2 and 3. Animals were maintained at 21°C ± 1°C and 55% humidity, under a 12‐h light/dark cycle, with irradiated chow. All experimental animals were male, and a heterozygous intercross strategy was employed to generate experimental mice/rats with defined genetic backgrounds and littermate controls.

Male Sprague–Dawley rats weighing 310–350 g at 8 weeks of age were used to establish a myosteatosis disease model. The high‐fat diet (HFD) group received a diet consisting of 60% energy from fat (a combination of lard and soybean oil), 20% energy from carbohydrates and 20% energy from protein (Daitz Biotech (Wuxi) Co. Ltd.; Product Code: HF60), while the control (CON) group received a regular chow diet (10% kcal from fat, 70% kcal from carbohydrates, and 20% kcal from protein; Dyets, LF10C) for 8 weeks. For the high‐fructose drinking water (HFru) group, 20% high‐fructose (Sigma, F3510) drinking water was provided, whereas the CON group received distilled water. Both groups had free access to water for 16 weeks.

For AdipoRon and fenofibrate administration, 6‐month‐old mice weighing 28–35 g were randomly divided into four groups as follows: WT + con (solvent control), KAL‐TG + con (solvent control), KAL‐TG + Adi (AdipoRon administration) and KAL‐TG + Feno (fenofibrate administration). A uniform suspension of AdipoRon and fenofibrate was prepared by dissolving the drug powder in 0.5% carboxymethyl cellulose sodium (CMC‐Na) and sonicating. Mice in the AdipoRon and fenofibrate treatment groups were given doses of 30 and 50 mg/kg/d, respectively. The other groups received equivalent volumes of the 0.5% CMC‐Na vehicle by oral gavage. After 2 weeks of continuous administration, exercise endurance was assessed.

### 2.2 Cell Culture Models

HEK293T cells (ATCC, Cat# CRL‐3216, RRID: CVCL_0063) were purchased from ATCC and C2C12 cells (CSTR:19375.09.3101HUMSCSP502, RRID: CVCL_0188) were kindly provided by the Stem Cell Bank, Chinese Academy of Sciences. Cells were cultured in Dulbecco's Modified Eagle's Medium (DMEM) (Gibco) supplemented with 10% fetal bovine serum (Gibco) and 1% streptomycin/penicillin (Hyclone) in a humidified incubator with 5% CO_2_ at 37°C. All cell lines have been validated by STR (Short Tandem Repeat) and tested negative for mycoplasma. For differentiation, C2C12 cells were switched to DMEM containing 2% horse serum (Biological Industries) and 1% streptomycin/penicillin, and differentiation was completed after 4 days.

### 2.3 Statistical Analysis

All statistical analyses were performed using GraphPad Prism 8 software. Data are presented as mean ± standard deviation. The *p* values were calculated using one‐way analysis of variance (ANOVA) with Tukey's post hoc test, repeated‐measures ANOVA with Dunnett's post hoc test, Kruskal–Wallis with Dunn's post hoc test or Student's *t*‐test, as indicated. Statistical significance is indicated as *p < 0.05, **p < 0.01 and ***p < 0.001 or ****p < 0.0001. ns, no significant differences were observed.

Additional materials and methods are provided in the Supporting Information.

## 3 Results

### 3.1 KAL Promotes Myosteatosis and Impairs Muscle Exercise Capacity

**FIGURE 1:**
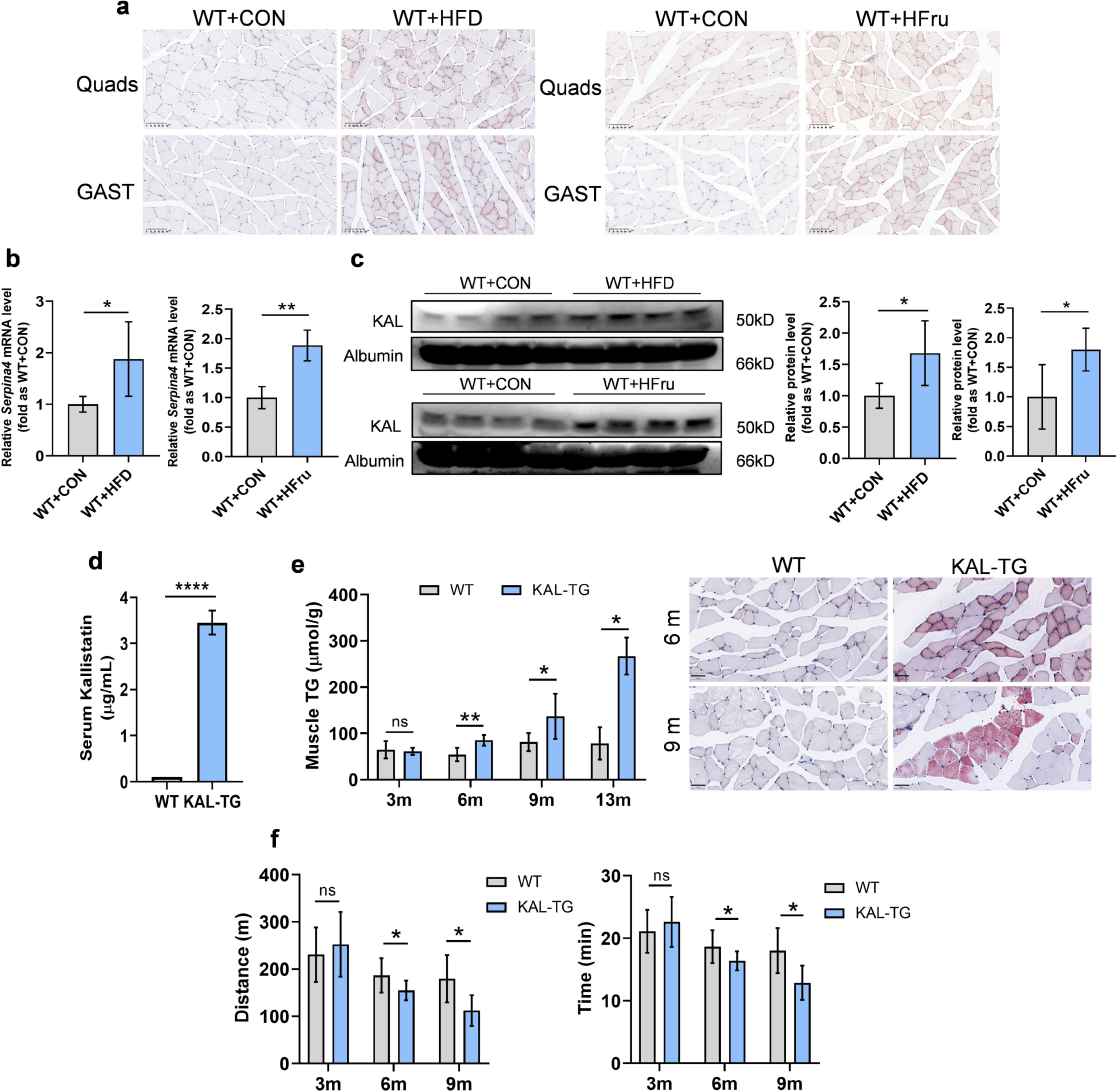
**An abnormally elevated KAL contributes to intramuscular lipid accumulation and reduced exercise capacity**. **(a)** Representative Oil Red O staining of rat muscle after 8 weeks of high‐fat diet and 16 weeks of high‐fructose drinking. Scale bars, 100 μm. **(b)** Transcription levels of rat liver *Serpina4* after high‐fat diet (*n* = 5) or high‐fructose drinking (*n* = 4). (c) Immunoblot and quantification of serum KAL after high‐fat feeding and high‐fructose intake (*n* = 4). (d) Serum KAL protein levels in WT and KAL‐TG mice (*n* = 4). (e) TG content in gastrocnemius across various age groups of mice (3‐, 6‐, and 9‐month‐old groups, *n* = 6 each; 13‐month‐old group, *n* = 3) and representative Oil Red O staining of gastrocnemius muscle from 6‐month‐old and 9‐month‐old mice. Normalization of triglyceride content in muscles based on protein concentration. Scale bars, 50 μm. **(f)** Age‐dependent alterations in exercise performance parameters as assessed by treadmill endurance testing (3‐month‐old groups, *n* = 5; 6‐month‐old groups, *n* = 8; 9‐month‐old group, *n* = 6). GAST, gastrocnemius; Quads, quadriceps; TG, triglyceride. *p < 0.05, **p < 0.01 and ****p < 0.0001. ns, no significant differences were observed.

Myosteatosis—pathological muscle lipid deposition—was induced in 8‐week‐old rats through established HFD (8 weeks) or 20% high‐fructose water (HFru; 16 weeks) protocols (S1–2). In our study, 8‐week‐old rats were either fed a HFD for 8 weeks or given 20% high‐fructose water for 16 weeks. Both regimens triggered significant lipid accumulation in myofibers (Figure 1a). Given KAL's hepatic origin, we quantified liver KAL transcription via qPCR. HFD and HFru robustly upregulated hepatic KAL mRNA (Figure 1b). This aligned with increased hepatic KAL expression in diabetic/MASLD patients (Figure S1a,b). Using immunoblotting (due to the unavailability of rat KAL ELISA kits), we confirmed elevated serum KAL levels in both models (Figure 1c), implicating KAL in the pathogenesis of myosteatosis.

To further elucidate the direct effects of KAL on muscle lipid content and exercise function, we generated KAL‐TG mice, which had significantly higher serum KAL levels than WT mice (Figures S1c and 1d). Our study revealed that KAL did not significantly affect the body weight or muscle weight of the mice (Figure S1d–g). However, compared with WT mice, KAL‐TG mice showed increased muscle TG content and lipid deposition from 6 months of age (Figure 1e). Moreover, the muscle‐FFA content of KAL‐TG mice did not show a significant increase until 13 months of age, while the TC content remained unchanged (Figure S2a–d). Importantly, no significant changes were observed in the circulating lipid levels of KAL‐TG mice, thereby excluding the potential confounding effect of dyslipidemia on muscle lipid accumulation (Figure S2e–h). Subsequent treadmill running assay revealed that KAL‐TG mice also exhibited exercise intolerance from 6 months of age compared with WT mice (Figure 1f). Moreover, muscle ATP content was reduced in KAL‐TG mice from this same age (Figure S2i). The phenotypic progression of KAL‐TG mice is summarized in Figure S2j.

**FIGURE 2:**
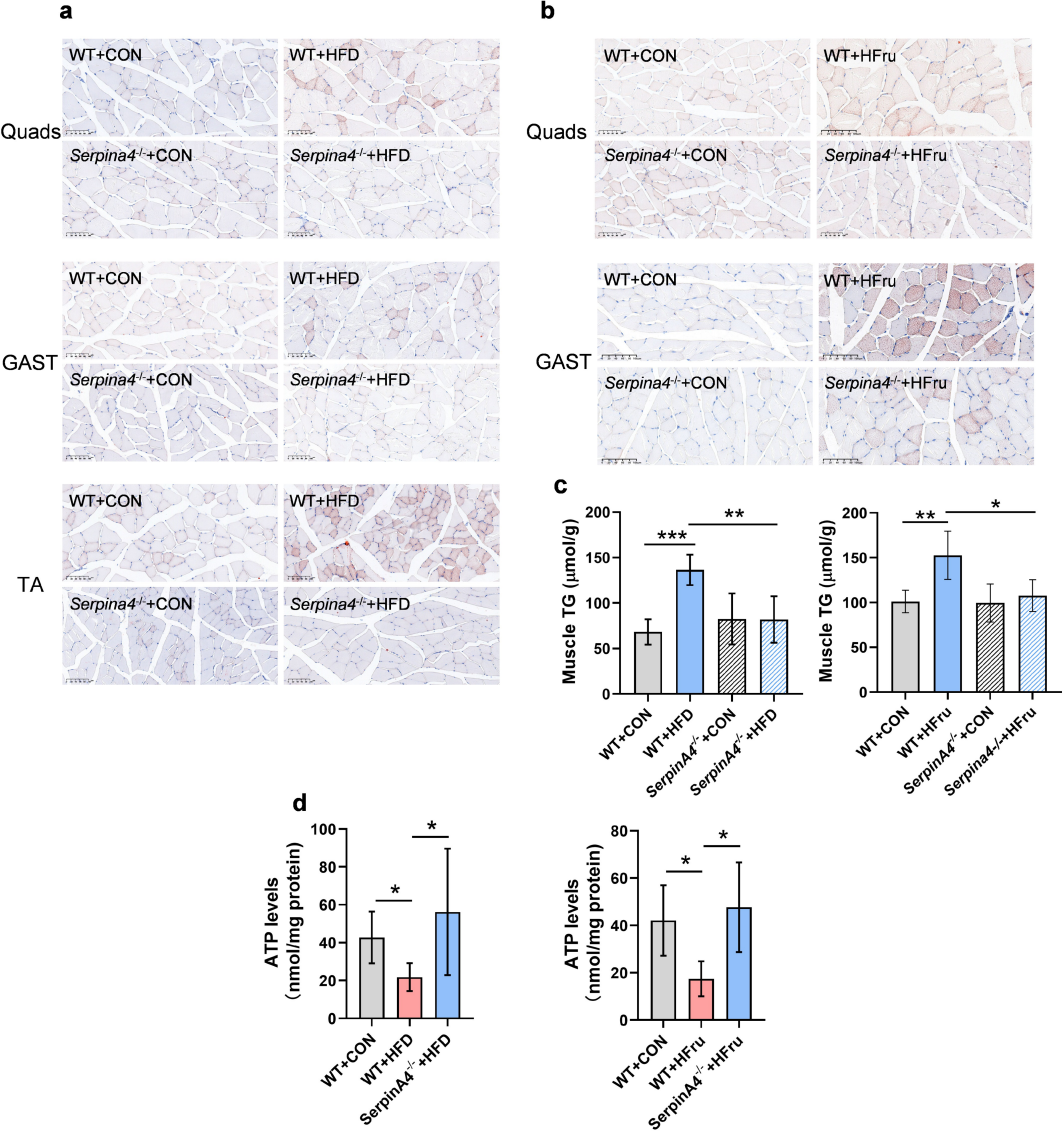
**Knocking out KAL has therapeutic effects in a myosteatosis model. (a)** Representative Oil Red O staining of muscle tissue in WT and KAL knockout rats following a high‐fat diet. Scale bars, 100 μm. **(b)** Representative Oil Red O staining of muscle tissue in WT and KAL knockout rats after high‐fructose intake. Scale bars, 100 μm. **(c)** Triglyceride (TG) content in the gastrocnemius muscle of WT and KAL knockout rats after a high‐fat diet (*n* = 6) and high‐fructose intake (*n* = 5). Normalization of triglyceride content in muscles based on protein concentration. **(d)** ATP levels in the gastrocnemius muscle of WT and KAL knockout rats were measured after a high‐fat diet (*n* = 6) and high‐fructose intake (*n* = 5). *p < 0.05, **p < 0.01 and ***p < 0.001.

Conversely, KAL knockout effectively ameliorated diet‐induced myosteatosis, suppressing intramuscular lipid deposition (Figures S2 and 2a,b) and reducing triglyceride accumulation (Figure 2c) in both high‐fat and high‐fructose models. Concomitantly, muscle ATP levels were restored following KAL ablation (Figure 2d), indicating recovery of cellular energy homeostasis. These findings demonstrate that pathological KAL elevation drives muscular metabolic dysfunction and exercise impairment.

### 3.2 KAL Promotes Myosteatosis via AMPK/ACC‐Mediated Lipogenesis

**FIGURE 3:**
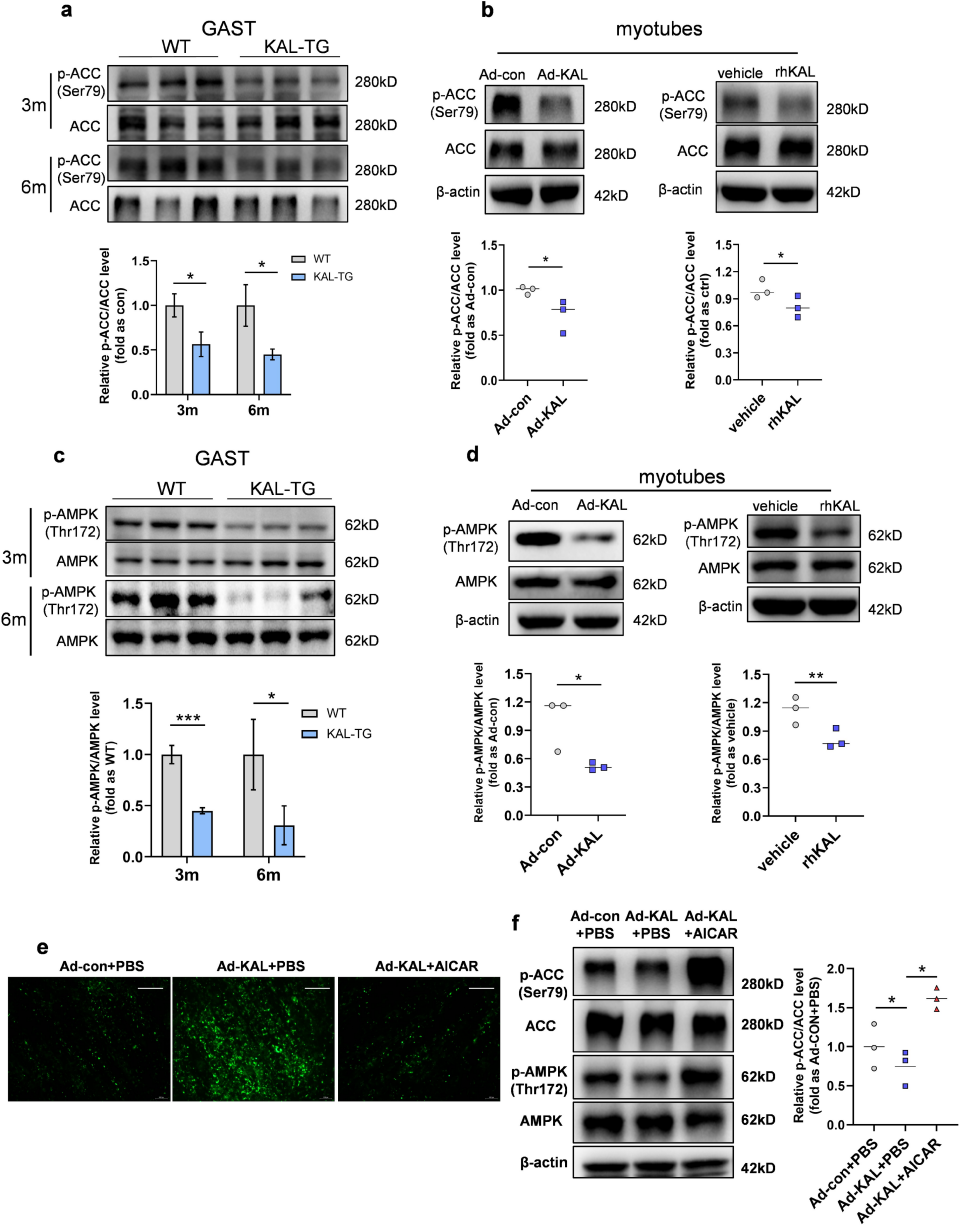
**KAL enhances de novo lipogenesis in skeletal muscle by inhibiting the AMPK/ACC pathway**. (**a**) Representative western blot analysis and quantification of p‐ACC/ACC levels in skeletal muscle from 3‐month‐old and 6‐month‐old mice. (**b**) Representative western blot and quantification of p‐ACC/ACC in myotubes treated with KAL adenovirus for 48 h or 0.5 μg/mL KAL recombinant protein (rhKAL) for 24 h. Each point represents an individual experiment (*n* = 3). (**c**) Representative western blot and quantification of p‐AMPK/AMPK in 3‐ month‐old and 6‐month‐old mice muscle. (**d**) Representative western blot and quantification of p‐AMPK/AMPK in myotubes treated with KAL adenovirus for 48 h or 0.5 μg/mL rhKAL for 24 h. Each point represents an individual experiment (*n* = 3). (**e**) Representative BODIPY staining of lipid accumulation in adenovirus‐treated myotubes. Scale bars, 100 μm. (**f**) Representative western blot and quantification of p‐AMPK/AMPK and p‐ACC/ACC in myotubes treated with 48 h adenovirus followed by 0.5 mM AICAR for 16 h. Each point represents an individual experiment (*n* = 3). *p < 0.05, **p < 0.01 and ***p < 0.001.

To investigate the mechanisms of KAL‐induced myosteatosis, we assessed the regulators of lipid metabolism. The in vitro results showed that overexpression of KAL did not significantly affect fatty acid uptake in myotubes (Figure S3a,b). No significant differences were observed in the expression levels of fatty acid uptake proteins, such as CD36 and FATP4, or lipolysis enzymes, including HSL, ATGL and CGI58, between WT and KAL‐TG mice (Figure S3c,d). However, ACC phosphorylation significantly decreased in the muscles of KAL‐TG mice at 3 and 6 months of age (Figure 3a), which indicated that enzyme activity may be elevated. By 13 months of age, ACC phosphorylation exhibited a sustained decrease, accompanied by an upregulation in FASN expression (Figure S3e). In vitro, lipid droplet accumulation in myotubes was observed following KAL overexpression (Figure S3f). Both KAL adenovirus transduction and recombinant human KAL protein (rhKAL) administration inhibited ACC phosphorylation in myotubes (Figure 3b). Excitingly, although the phosphorylation of ACC significantly decreased after a HFD, it was effectively replenished after knocking out KAL (Figure S3g). These findings demonstrate that KAL inhibits ACC phosphorylation, thereby promoting de novo lipogenesis in skeletal muscle. Importantly, our study reveals that this de novo lipogenesis pathway makes a substantial contribution to the development of intramyocellular lipid accumulation.

As AMPK phosphorylates and inhibits ACC, we assessed AMPK signalling. The results demonstrated a significant reduction in AMPK phosphorylation levels in the muscles of 3‐month‐old KAL‐TG mice compared to WT mice, with a further decrease observed at 6 months of age (Figure 3c). Furthermore, in vitro experiments showed that overexpression of KAL or rhKAL treatment significantly reduced AMPK phosphorylation in myotubes (Figure 3d). In animal disease models in vivo, high‐fat or high‐fructose intake inhibits muscle AMPK phosphorylation, and knocking out KAL significantly restores it (Figure S3h). AICAR‐mediated AMPK reactivation blocked KAL‐induced lipid accumulation and ACC dephosphorylation (Figure 3e,f). Thus, KAL inhibits AMPK, reducing ACC phosphorylation and enhancing lipogenesis.

### 3.3 KAL Reduces Skeletal Muscle Mitochondrial Content and Decreases Oxidative Energy Supply

**FIGURE 4:**
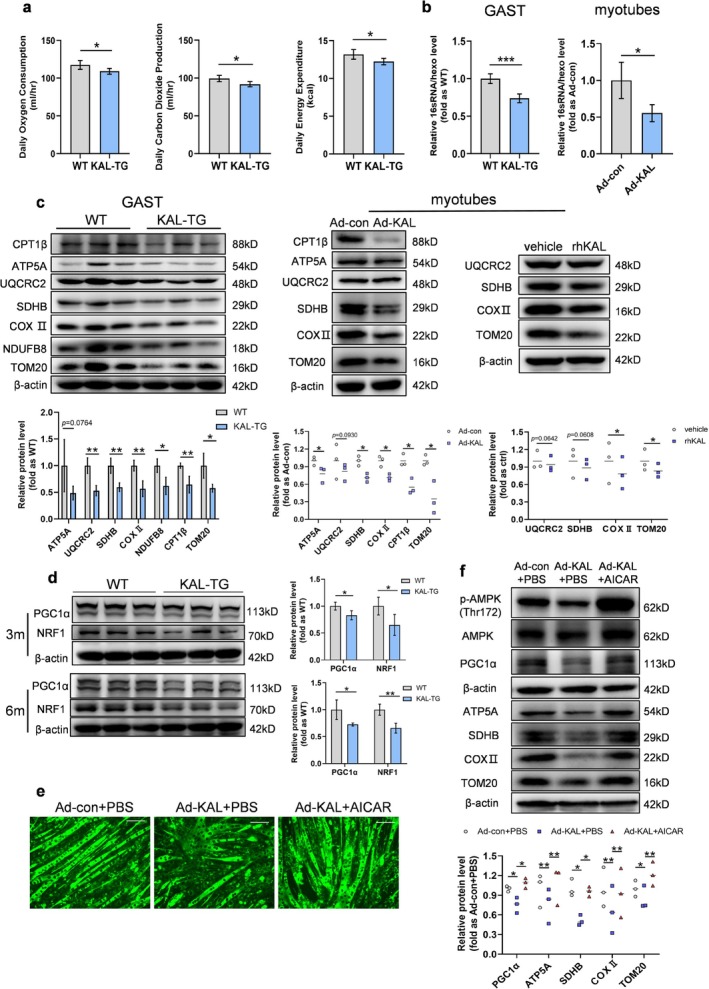
**KAL leads to a decrease in muscle energy supply through inhibiting mitochondrial biogenesis.** (**a**) Metabolic parameters in 6‐month‐old mice: maximal oxygen consumption (VO_2_max), carbon dioxide production (VCO_2_), and energy expenditure (*n* = 4). (**b**) Mitochondrial DNA copy number in the gastrocnemius of 6‐month‐old mice and adenovirus‐treated myotubes (*n* = 4). (**c**) Representative immunoblots and quantification of gastrocnemius CPT1β, oxidative phosphorylation (OXPHOS)‐related proteins, and TOM20 in 6‐month‐old mice and myotubes treated with adenovirus for 48 h or 0.5 μg/mL rhKAL for 24 h (*n* = 3). Each point represents an individual experiment. (**d**) Protein levels of PGC1α and NRF1 in gastrocnemius of 3‐ month‐old and 6‐month‐old mice. (**e**) Representative Mitotracker staining of myotubes treated with 48 h adenovirus followed by 0.5 mM AICAR for 16 h. Scale bars, 100 μm. (**f**) Representative immunoblot and quantification of p‐AMPK/AMPK, OXPHOS‐related proteins, TOM20 and PGC1α in myotubes treated with 48 h adenovirus followed by 0.5 mM AICAR for 16 h (*n* = 3). Each point represents an individual experiment. *p < 0.05, **p < 0.01 and ***p < 0.001.

Mitochondria serve as a crucial intracellular site for oxidative energy production, which powers muscle movement. Consistent with unaltered exercise endurance at 3 months, muscle ATP and mitochondrial content did not differ between WT and KAL‐TG mice (Figures S2i and S4a,b). However, maximum oxygen consumption, carbon dioxide production and energy expenditure were significantly lower in KAL‐TG mice compared to WT mice at 6 months of age (Figure 4a). Additionally, there was no significant difference in food intake (Figure S4c), which rules out the influence of dietary differences. Moreover, mitochondrial and ATP content was significantly reduced by excess KAL (Figures 4b,c and S4d–f). Furthermore, knocking out KAL in vivo effectively inhibited the decrease in muscle mitochondrial number caused by high‐fat or high‐fructose intake (Figure S4g). The findings show that KAL reduces mitochondrial content in muscles, thereby compromising the energy supply during exercise.

Maintaining high‐quality and normal mitochondrial content in cells depends on the balance between autophagy and mitochondrial biogenesis, which controls the amount of mitochondria in cells. The results showed that KAL overexpression markedly reduced the transcription levels of *Ppargc1a, Tfam*, *Nrf1* and *Nrf2* in the muscles of 3‐month‐old mice (Figure S5a), along with a corresponding decrease in PGC‐1α and NRF1 protein expression (Figure 4d). Moreover, consistent with in vivo findings, both KAL overexpression and rhKAL protein treatment decreased PGC‐1α and NRF1 expression in myotubes (Figure S5b,c). However, no changes in autophagy were observed in vivo or in vitro (Figure S5d,e). As AMPK regulates PGC‐1α [19], reactivation of AMPK via AICAR rescued KAL‐induced biogenesis defects (Figure 4e,f). Collectively, these findings suggest that KAL suppresses mitochondrial biogenesis by downregulating the AMPK/PGC‐1α/NRF1 signalling axis.

### 3.4 KAL Binds to AdipoR1 and Inhibits the AMPK Upstream Kinases LKB1 and CaMKK2

**FIGURE 5:**
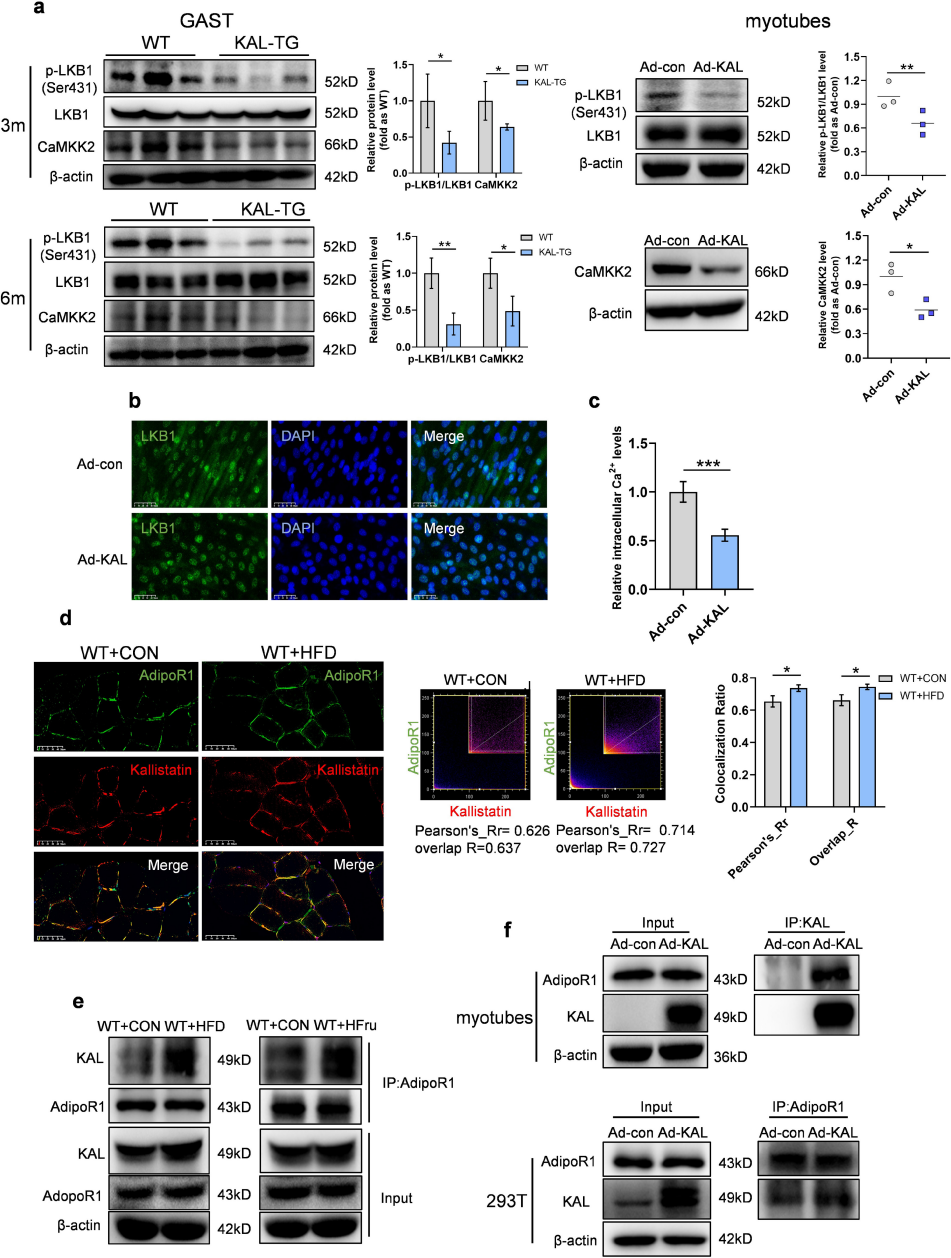
**KAL can inhibit LKB1/CaMKK2 and bind to AdipoR1 on the sarcolemma.** (**a**) Representative immunoblot analysis and quantification of p‐LKB1/LKB1 and CaMKK2 expression in gastrocnemius from 3‐ month‐old and 6‐month‐old mice and adenovirus‐treated myotubes (*n* = 3). Each point represents an individual experiment. (**b**) Representative immunofluorescence staining of LKB1 in adenovirus‐treated myotubes. Scale bars, 50 μm. (**c**) Representative intracellular Ca^2+^ concentration in adenovirus‐treated myotubes (*n* = 4). (**d**) Representative co‐immunofluorescence staining using anti‐AdipoR1 (green) and anti‐KAL (red) in gastrocnemius. Scale bars, 50 μm. Scatter plot showing co‐localization of KAL and AdipoR1 from the co‐immunofluorescence staining. Pearson's correlation coefficient and overlap coefficient were used to analyse the KAL‐AdipoR1 colocalization (*n* = 3). (**e**) Co‐immunoprecipitation of KAL and AdipoR1 from gastrocnemius. (**f**) Representative co‐immunoprecipitation of KAL and AdipoR1 in differentiated myotubes and 293 T cells. *Note:* Primary antibodies for IP and immunoblotting were derived from different species. *p < 0.05, **p < 0.01 and ***p < 0.001.

The activation of AMPK depends on phosphorylation of Thr172 on the alpha subunit and is regulated by various upstream kinases, including LKB1 and CaMKK2. In KAL‐TG mice, skeletal muscle exhibited significantly reduced phosphorylation of LKB1‐Ser431 and lower CaMKK2 levels (Figure 5a). Similarly, KAL overexpression in myotubes decreased LKB1‐Ser431 phosphorylation, cytoplasmic localization and Ca^2+^ levels (Figure 5a–c). While phosphatases or PKA/AKT‐mediated phosphorylation can inhibit AMPK [20, 21], their expression and activity were unchanged in KAL‐TG muscle (Figure S6a). Thus, KAL primarily suppresses AMPK phosphorylation via impaired LKB1 and CaMKK2 signalling.

As KAL signals extracellularly via receptors, we assessed LRP6, a known KAL receptor and Wnt coreceptor [22]. Given low LRP6 expression in skeletal muscle [23], neither β‐catenin activity (Figure S7a) nor LRP6 lacking (Figure S7b) altered KAL‐mediated AMPK inhibition, excluding LRP6 involvement.

AdipoR1, highly expressed in skeletal muscle, primarily activates AMPK. Its main pathway for activating AMPK is to promote LKB1 cytoplasmic translocation via the intracellular adaptor protein APPL1, with a secondary pathway increasing intracellular Ca^2+^ and CaMKK2 [24, 25]. Moreover, the expression level of AdipoR1 in muscle is significantly higher than that of LRP6 (Figure S7c). Therefore, we hypothesized that KAL inhibits AMPK by binding to AdipoR1. H‐dock and PPA tools were used for protein‐binding prediction (S3–4). The results showed that the docking score, Kd value and ΔG of KAL‐AdipoR1 were lower than those of KAL‐LRP6, suggesting stronger KAL‐AdipoR1 binding affinity than KAL‐LRP6 (Figure S7d). Immunofluorescence analysis of muscle tissue revealed that KAL co‐localized with AdipoR1 and the co‐localization coefficients of the two in the high‐fat‐fed group were significantly higher than those in the CON group (Figure 5d). Co‐immunoprecipitation in muscle and cells verified this interaction (Figure 5e,f), indicating KAL signals through sarcolemmal AdipoR1.

### 3.5 KAL Relies on AdipoR1 to Suppress AMPK

**FIGURE 6:**
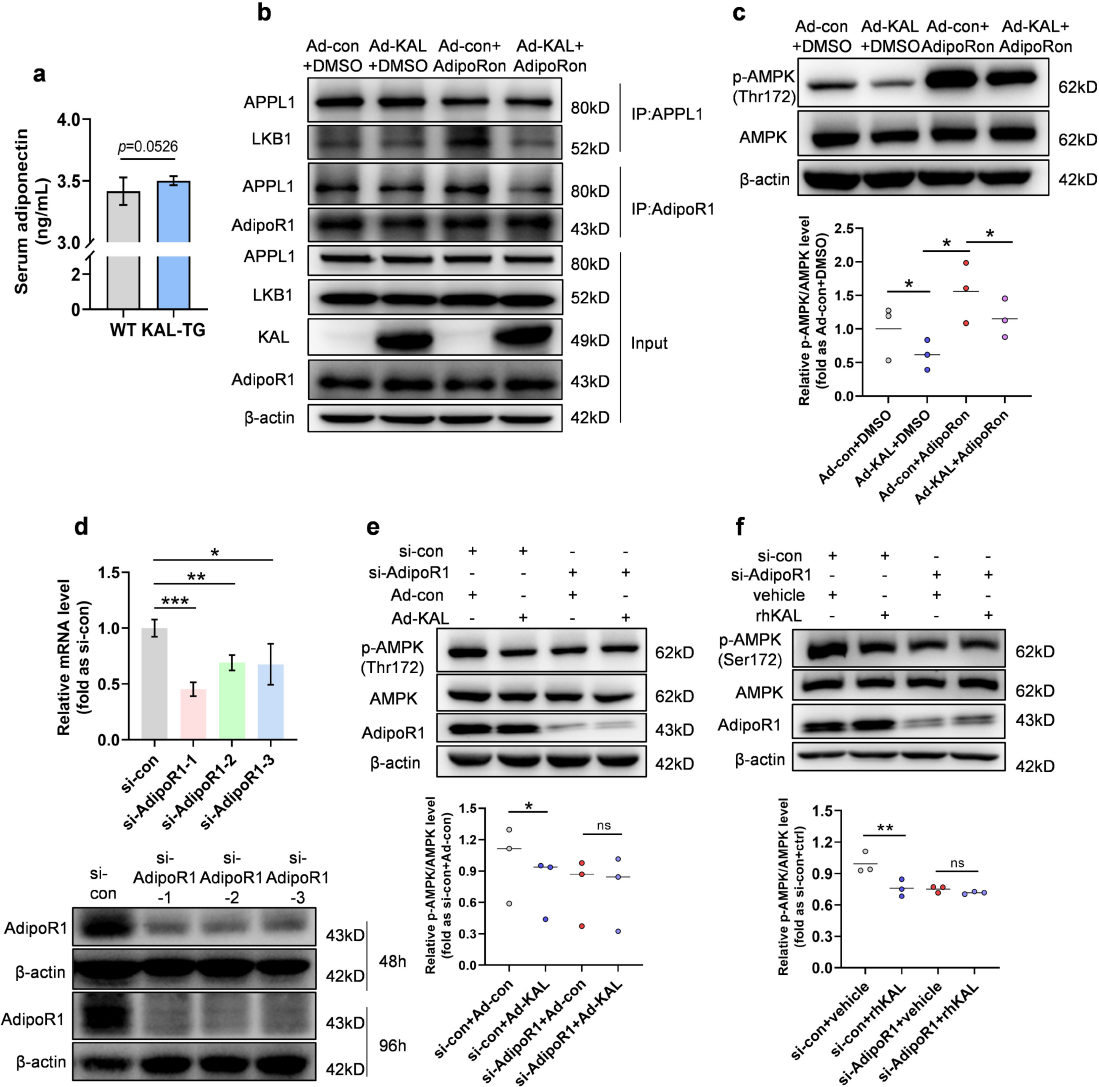
**KAL depends on AdipoR1 for the signal transmission. (a)** Serum adiponectin levels in 3‐month‐old mice (*n* = 6). (**b**) Representative co‐immunoprecipitation (IP) analysis of KAL/AdipoR1 and APPL1/AdipoR1 interactions in myotubes treated with 48 h adenovirus followed by 50 μM AdipoRon for 90 min. (**c**) Representative immunoblots and quantitative analysis of p‐AMPK/AMPK levels in myotubes treated with 48 h adenovirus followed by 50 μM AdipoRon for 90 min (*n* = 3). Each point represents an individual experiment. (**d**) AdipoR1 mRNA and protein expression in myotubes following siRNA‐mediated knockdown (three distinct siRNAs tested). (**e**) Representative immunoblotting and quantitative analysis of p‐AMPK/AMPK in AdipoR1‐knockdown myotubes overexpressing KAL (*n* = 3). Each point represents an individual experiment. (**f**) Immunoblots and quantification of p‐AMPK/AMPK in AdipoR1‐knockdown myotubes treated with recombinant KAL protein (*n* = 3). Each point represents an individual experiment. *Note:* Primary antibodies for IP and immunoblotting were derived from different species. *p < 0.05, **p < 0.01 and ***p < 0.001. ns, no significant differences were observed.

To determine whether KAL‐AdipoR1 interaction modulates AdipoR1‐AMPK signalling, we first assessed key pathway components. There was no notable difference in muscle AdipoR1 expression (Figure S7e,f) or serum adiponectin levels (Figure 6a) between the WT and KAL‐TG mice. Furthermore, neither adiponectin nor intracellular adaptor protein APPL1 in the muscles of 3‐month‐old mice showed a significant difference between KAL‐TG and WT mice (Figure S7g). These results indicate that KAL does not suppress AMPK by reducing adiponectin/AdipoR1/APPL1 expression.

Further immunoprecipitation assays revealed that KAL overexpression attenuated interactions between AdipoR1 and APPL1, as well as between APPL1 and LKB1, in myotubes following AdipoRon treatment, an AdipoR1 agonist (Figure 6b). The results revealed that AdipoRon robustly activated AMPK in myotubes, whereas KAL partially inhibited this response (Figure 6c). Three different AdipoR1 siRNAs were constructed, all of which exhibited efficient knockdown effects 48–96 h after transfection (Figure 6d; si‐AdipoR1‐1 selected for strongest silencing). We subsequently overexpressed KAL following AdipoR1 knockdown in myotubes, and the results indicated that KAL's inhibitory effect on AMPK was effectively blocked after AdipoR1 knockdown (Figure 6e). Similarly, in myotubes lacking AdipoR1, the rhKAL protein lost its inhibitory effect on AMPK (Figure 6f). These data demonstrate that KAL's inhibitory effect on AMPK in myotubes depends on AdipoR1, which inhibits AdipoR1‐AMPK signalling.

### 3.6 AdipoRon and Fenofibrate Improve KAL‐Induced Impaired Exercise Capacity and Myosteatosis

**FIGURE 7:**
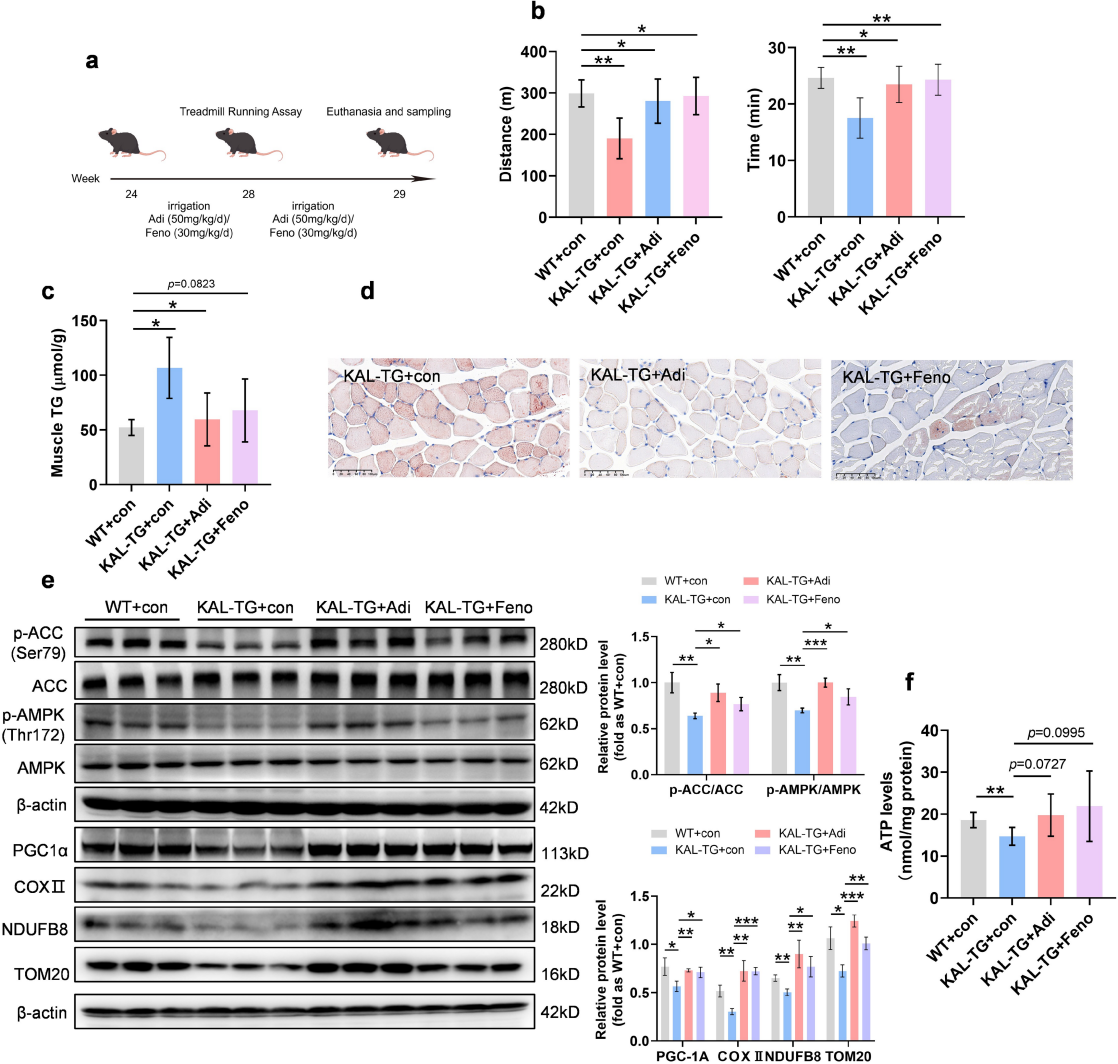
**AdipoRon and fenofibrate can improve exercise intolerance and myosteatosis caused by KAL.** (**a**) AdipoRon and fenofibrate gavage administration timeline. (**b**) The maximum running distance and duration of WT, KAL‐TG, KAL‐TG + Adi (AdipoRon administration) and KAL‐TG + Feno mice (fenofibrate administration) (*n* = 5). (**c**) Triglyceride (TG) content in gastrocnemius of WT, KAL‐TG, KAL‐TG + Adi, and KAL‐TG + Feno mice (*n* = 5). Normalization of triglyceride content in muscles based on protein concentration. (**d**) Representative Oil red staining of quadriceps in KAL‐TG, KAL‐TG + Adi, and KAL‐TG + Feno mice. Scale bars, 100 μm. (**e**) Representative immunoblot and quantification of p‐AMPK/AMPK, p‐ACC/ACC, PGC1 α, COX II, NDUFB8, and TOM20 in gastrocnemius of WT, KAL‐TG, KAL‐TG + Adi and KAL‐TG + Feno mice. (**f**) Relative ATP level in gastrocnemius of WT, KAL‐TG, KAL‐TG + Adi and KAL‐TG + Feno mice (*n* = 5). *p < 0.05, **p < 0.01 and ***p < 0.001. ns, no significant differences were observed.

To date, no drugs targeting AdipoR have been approved for clinical use. The development of small‐molecule peptides derived from adiponectin has emerged as a key area of research for the treatment of metabolic diseases. AdipoRon, as a small‐molecule AdipoR1 agonist with oral bioavailability, has potential clinical applications [26]. Beyond its function as a peroxisome proliferator‐activated receptor‐alpha (PPAR‐α) agonist and lipid‐lowering agent, our previous work demonstrates that fenofibrate inhibits KAL expression in liver cells [16, 27]. Therefore, further experiments were designed to verify whether in vivo administration of AdipoRon and fenofibrate could alleviate myosteatosis and exercise intolerance caused by abnormally elevated KAL. Six‐month‐old KAL‐TG mice were gavaged with AdipoRon (50 mg/kg/d) or fenofibrate (30 mg/kg/d) for 4 weeks (Figure 7a). The results of the exercise endurance test showed that the total distance and duration of exercise in KAL‐TG mice were significantly increased after administration of these two agents (Figure 7b). The lipid content in the muscle of KAL‐TG mice was reduced after AdipoRon and fenofibrate administration (Figure 7c,d). After administering AdipoRon or fenofibrate, the phosphorylation levels of AMPK and ACC in the muscles of KAL‐TG mice were restored (Figure 7e). Subsequent experiments demonstrated a marked increase in ATP production and mitochondrial content in muscle tissue following administration of AdipoRon and fenofibrate (Figure 7e,f). These data suggest that repurposing AdipoR1 agonists (e.g., AdipoRon) or reducing circulating KAL levels (e.g., fenofibrate) may represent therapeutic strategies for myosteatosis and exercise intolerance in diabetes and MASLD.

## 4 Discussion

**FIGURE 8:**
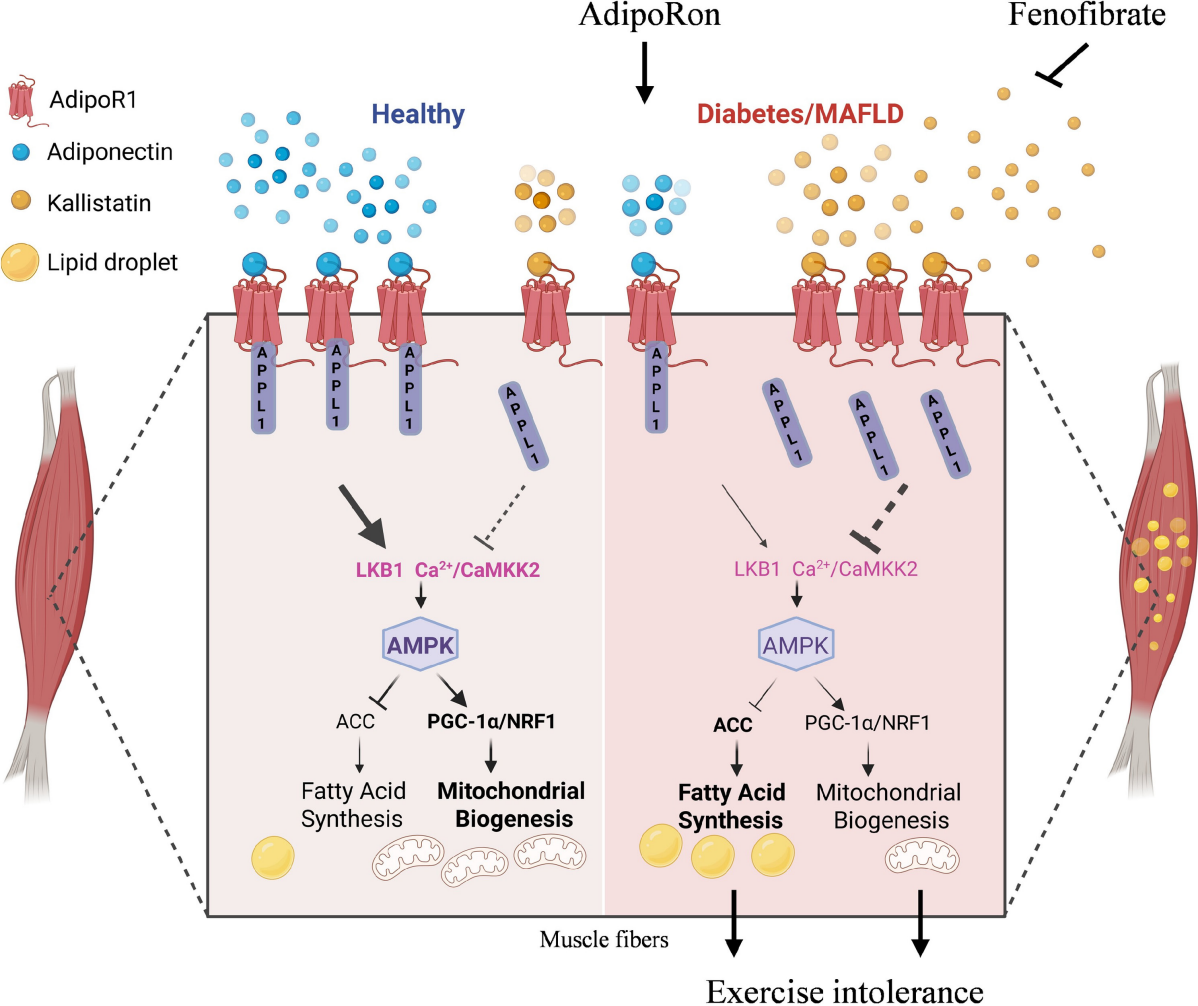
Kallistatin modulates AMPK activity by suppressing both LKB1 and Ca^2+^/CaMKK2 pathways through its novel target—AdipoR1, thereby promoting intramuscular lipid deposition and exercise intolerance. A proposed working model delineates the mechanism through which KAL exacerbates metabolic dysfunction in muscle cells. Specifically, KAL binds to AdipoR1 on the sarcolemmal, impairs APPL1‐mediated cytoplasmic translocation of LKB1, and reduces cytoplasmic Ca^2+^/CaMKK2 levels. Consequently, these changes lead to inadequate AMPK activation, decreased ACC phosphorylation, and downregulation of mitochondrial biogenesis genes PGC1α and NRF1. The increased ACC activity contributes to lipid accumulation in muscle, while impaired mitochondrial gene expression results in insufficient energy production, thereby synergistically contributing to exercise‐related injuries. Consequently, there are two strategies: repurposing AdipoR1 agonists (e.g., AdipoRon) or reducing circulating KAL (e.g., genetic ablation, triglyceride‐lowering agents such as fenofibrate), both applicable to diabetes/MASLD‐related metabolic myopathy. (Created with bioRender.com).

In summary, our observations revealed an underappreciated role of aberrantly elevated KAL in the de novo lipogenesis and mitochondrial biogenesis in muscle, contributing to myosteatosis and impaired exercise capacity (Figure 8). Mechanistically, we elucidated that KAL induces myosteatosis and exercise intolerance by inhibiting the AdipoR1‐AMPK axis. The subsequently inhibited AMPK exerts dual effects: it inhibits ACC phosphorylation while concurrently downregulating PGC1α and NRF1. Phenotypic analysis of KAL‐TG mice revealed a continuous and significant increase in muscle triglyceride content starting at 6 months of age (Figure 1e), whereas fatty acid levels do not exhibit a significant rise until 13 months (Figure S2). This temporal discrepancy may be attributed to an initial downregulation of ACC phosphorylation, which enhances its activity and leads to elevated TG accumulation (Figure 3a). Beyond 6 months of age, the concurrent decrease in ACC phosphorylation, reduction in mitochondrial content and increase in FASN expression by 13 months further exacerbated lipid metabolism dysregulation in muscle tissue (Figures 4a–c and S3e). Myosteatosis and reduced mitochondrial content emerged concurrently at 6 months of age in KAL‐TG mice, both of which likely contribute synergistically to the development of exercise intolerance. This decline in exercise capacity occurred without accompanying muscle mass loss (Figure S1), indicating that KAL induces dynamic skeletal muscle dysfunction rather than sarcopenic muscle atrophy.

Accumulating evidence supports KAL as a promising therapeutic target for metabolic diseases. Our team, along with other research groups, has identified KAL as a potential diagnostic biomarker in diabetes [17, 28]. Notably, both type I and type II diabetes patients exhibit significantly elevated serum KAL levels [28, 29]. Our preliminary studies further demonstrate that MASLD patients exhibit increased serum KAL concentrations, which positively correlate with TG, FFA, TC and LDL levels, while inversely correlating with HDL [16]. The findings of other research teams align with our results [30]. Chinese researchers have constructed a comprehensive human health and disease plasma proteome map using over 50 000 samples from NHANES, revealing the relationship between circulating proteins and various diseases [31]. Analysis based on this plasma proteome map demonstrated that KAL is a risk factor for HLP, nonalcoholic fatty liver disease (also known as MASLD), diabetes and obesity. However, in metabolically healthy African American adolescents, plasma KAL levels were inversely associated with obesity, adverse lipid profiles and inflammation [32]. The metabolic effects of KAL may vary depending on genetic background, physiopathological status, disease stage or differences in fat distribution within the population. This variation may be mediated by opposing signalling pathways in muscle and adipose tissue, which needs to be verified by subsequent studies.

While skeletal muscle is primarily regarded as an energy‐consuming organ, the pathological role of de novo lipogenesis in muscle lipid accumulation remains a controversial and understudied topic. In this study, we present novel evidence that inhibited ACC phosphorylation and subsequent increased de novo lipogenesis in myotubes constitute a significant pathological mechanism in the development of myosteatosis (Figure 3). Here, KAL primarily induced a significant elevation in muscle TG levels (Figure 1e). Intramuscular lipids are predominantly composed of triglycerides (IMTG), with ATGL and HSL responsible for approximately 98% of muscle TG hydrolysis [33, 34]. However, our study revealed that KAL did not affect ATGL, HSL or even CGI58 (Figure S3c,d), which contrasts with our previous findings in the liver [16]. The observed differential signalling is likely attributable to tissue‐specific pathway utilization, whereby KAL primarily engages LRP6‐mediated signalling in the liver and relies on AdipoR1 as its main signalling mediator in skeletal muscle.

The receptor preference of KAL appears to be tissue‐dependent. This specificity may be attributed to the predominant and broad expression of AdipoR1 in skeletal muscle (S5), where its level exceeds that of LRP6 (Figure S7c). Consequently, KAL is likely to transmit signals primarily through AdipoR1 in this tissue. Based on this, it provides a rational blueprint for developing organ‐optimized therapeutics. For metabolic diseases characterized by tissue‐specific pathology, such as myosteatosis in type 2 diabetes or hepatic steatosis in MASLD, one could design KAL analogs with enhanced affinity for AdipoR1 (for muscle‐targeted therapy) or LRP6 (for liver‐targeted therapy). Our findings suggest a viable strategy for combination therapy across multiple organs. Given the interconnected nature of metabolic disorders, a single agent or coordinated treatment approach that concurrently modulates both AdipoR1 and LRP6 pathways could ameliorate dysfunction across muscle and liver, thereby yielding superior systemic benefits.

The interaction between KAL and adiponectin signalling presents an intriguing regulatory mechanism. Adiponectin, the canonical AdipoR1 ligand, circulates at concentrations of 2–20 μg/mL (0.01%–0.05% of total serum protein) [35] and demonstrates well‐established metabolic benefits [36] (S6–7). However, its levels are markedly reduced in metabolic disorders (5.32 ± 1.47 μg/mL in diabetes [37]; 3.61 ± 1.31 μg/mL in MASLD [38]). In contrast, KAL levels are significantly elevated in these conditions (17.9 ± 13.8 μg/mL in diabetes [17]; 7.77 ± 1.16 μg/mL in MASLD [16]). Our discovery that KAL functions as an endogenous AdipoR1 antagonist suggests a novel regulatory axis in metabolic disease. Decreased adiponectin and increased KAL in T2DM/MASLD significantly suppress AdipoR1‐mediated AMPK activation in skeletal muscle. Pharmacological activation of AdipoR1 with adipoRon effectively reversed KAL‐induced myosteatosis and restored exercise capacity, highlighting the therapeutic potential of this pathway.

Fenofibrate is a PPAR‐α agonist with broad metabolic and pleiotropic effects (S8), including the reduction of muscular fat via the PPARα‐AMPK pathway [39]. Building on our prior observations that fenofibrate counteracts FFA‐induced KAL elevation [16, 27], we now demonstrate it antagonizes KAL‐driven myosteatosis and exercise intolerance (Figure 7). We propose that suppressing KAL and its downstream effects provides a complementary mechanism for fenofibrate in muscle, consistent with the improved phenotype seen in KAL knockout mice (Figure 2). Given that AMPK is inhibited downstream of KAL, activating AMPK presents a promising therapeutic strategy. Metformin, a known AMPK activator, has been shown in clinical studies to reduce muscle lipid content [40]. Notably, combining metformin with fenofibrate proves more effective against metabolic syndrome than either drug alone (S9), suggesting that similar combination therapy may benefit metabolic myopathy—potentially via enhanced AMPK activation. Extending this logic, the co‐administration of metformin and AdipoRon, both targeting AMPK, is mechanistically poised for synergistic effects. Systematic evaluation of such combination therapies represents a critical future direction.

Collectively, these findings indicate that KAL monoclonal antibodies, fenofibrate, AdipoRon and metformin all exhibit substantial therapeutic potential for addressing both myosteatosis and associated exercise intolerance in metabolic disorders.

## Conflicts of Interest

The authors declare no conflicts of interest.

## Supporting information




**Data S1:** Supplementary information.

**Table S1:** Interference sequence and primer sequence.
**Table S2:** Antibodies information.
**Table S3:** Reagent information.
**Table S3:** Triglyceride content in the gastrocnemius muscle of 3‐month‐old mice.
**Table S4:** Triglyceride content in the gastrocnemius muscle of 6‐month‐old mice.
**Table S5:** Triglyceride content in the gastrocnemius muscle of 9‐month‐old mice.
**Table S6:** Triglyceride content in the gastrocnemius muscle of 13‐month‐old mice.
**Table S7:** Triglyceride content in the gastrocnemius muscle of rats after control and high‐fat diet.
**Table S8:** Triglyceride content in the gastrocnemius muscle of rats after control and high‐fructose intake.
**Table S9:** Triglyceride content in the gastrocnemius muscle of rats after AdipoRon and fenofibrate treatment.
**Table S10:** The running distance of 3‐month‐old mice during exercise endurance experiments.
**Table S11:** The running distance of 6‐month‐old mice during exercise endurance experiments.
**Table S12:** The running distance of 9‐month‐old mice during exercise endurance experiments.
**Table S13:** The running distance of mice during exercise endurance experiments after AdipoRon and fenofibrate treatment.

**Figure S1:** jcsm70261‐sup‐0003‐Supplementary_Figures.pdf. **There is no significant difference in body weight and muscle weight between KAL‐TG and WT mice**. (**a–b**) *SERPINA4* mRNA levels in human liver tissues from GEO datasets GSE89632 (**a**) and GSE23343 (**b**). (**c**) Genotyping results of WT and KAL‐TG mice. (d–g) Body weight and muscle mass measurements in KAL‐TG and WT mice at 3 months (d), 6 months (e), 9 months (f) and 13 months (g) of age. 3‐, 6‐ and 9‐month‐old groups, *n* = 6 each; 13‐month‐old group, *n* = 3. *p < 0.05 and **p < 0.01. ns, no significant differences were observed.
**Figure S2: Changes in muscle lipids of KAL‐TG mice.** (**a–d**) Muscle cholesterol (CHO) and fatty acid (FA) content in 3‐month‐old (**a**), 6‐month‐old (**b**), 9‐month‐old (**c**) and 13‐month‐old (**d**) mice. 3‐, 6‐ and 9‐month‐old groups, *n* = 6 each; 13‐month‐old group, *n* = 3. (e–h) The levels of serum total cholesterol (TC), HDL‐C, LDL‐C, total triglycerides (TG) and free fatty acids (FFA) in mice at 3‐month‐old (e, *n* of WT = 4, *n* of KAL‐TG = 5), 6‐month‐old (f, *n* of WT = 9, *n* of KAL‐TG = 8), 9‐month‐old (g, *n* = 8) and 13‐month‐old (h, *n* of WT = 5, *n* of KAL‐TG = 7) mice. (i) ATP levels in the gastrocnemius from mice at different ages. 3‐, 6‐ and 9‐month‐old groups, *n* = 6 each; 13‐month‐old group, *n* = 3. (j) Chronological progression of phenotypic manifestations in KAL‐TG mice. (k) qPCR results of hepatic *Serpina4* (*n* = 5). *p < 0.05 and **p < 0.01. ns, no significant differences were observed.
**Figure S3: Pathological upregulation of KAL promotes aberrant de novo lipogenesis through AMPK.** (**a**) KAL levels in the culture supernatant of adenovirus‐treated myotubes (*n* = 3). (**b**) Relative fatty acid uptake in myotubes following adenovirus treatment (*n* = 5). (**c–d**) Representative immunoblot of lipid metabolism regulators in the gastrocnemius of 6‐month‐old mice: (**c**) CD36, FATP4 and p‐HSL/HSL; (**d**) FASN, ATGL and CGI58. (**e**) Representative immunoblots and quantification of p‐ACC/ACC and FASN in the gastrocnemius of 13‐month‐old mice. (**f**) Representative Oil Red O and BODIPY staining in adenovirus‐treated myotubes. Scale bars, 100 μm. (**g**) Representative western blot and quantification of p‐ACC/ACC in gastrocnemius muscle from high‐fat diet‐fed mice. (**h**) Representative immunoblotting and statistical analysis of p‐AMPK/AMPK in rat gastrocnemius muscle after high‐fat diet and high‐fructose intake. **p < 0.01, ***p < 0.001 or ****p < 0.0001. ns, no significant differences were observed.
**Figure S4: Pathologically elevated KAL reduces mitochondrial content.** (**a**) Mitochondrial DNA copy number in the gastrocnemius of 3‐month‐old mice (*n* = 3). (**b**) Representative immunoblot of oxidative phosphorylation (OXPHOS)‐related protein and TOM20 levels in the gastrocnemius of 3‐month‐old mice and quantitative analysis. (**c**) Food intake in 6‐month‐old mice (*n* = 4). (**d**) Representative immunoblot and quantitative analysis of OXPHOS‐related protein levels in the gastrocnemius muscle of 13‐month‐old mice. (**e**) ATP levels in adenovirus‐treated differentiated myotubes (*n* = 3). (**f**) Representative Mitotracker staining of mitochondria in myotubes treated with adenovirus for 48 h or 0.5 μg/mL rhKAL for 24 h. Scale bars, 100 μm. (**g**) Representative immunoblot and quantification of CPT1β, OXPHOS‐related proteins and TOM20 in gastrocnemius from rats after high‐fat diet or high‐fructose intake. *p < 0.05, **p < 0.01 and ***p < 0.001. ns, no significant differences were observed.
**Figure S5: KAL regulates mitochondrial biogenesis.** (**a**) mRNA expression levels of *Ppargc1α*, *Tfam*, *Nrf1* and *Nrf2* in the gastrocnemius muscle of 3‐month‐old mice (*n* = 4). (**b**) Representative immunoblot analysis of PGC1α and NRF1 in myotubes transduced with adenovirus and corresponding quantification (*n* = 3). Each point represents an individual experiment. (**c**) Representative immunoblots and quantification analysis of PGC1α and NRF1 in myotubes treated with rhKAL protein (*n* = 3). Each point represents an individual experiment. (**d**) LC3B‐II/LC3B‐I ratio in the gastrocnemius of 6‐month‐old mice, assessed by immunoblotting and quantified. (**e**) Representative immunoblots and statistical analysis of Parkin and LC3B‐II/LC3B‐I in cytoplasmic and mitochondrial fractions of adenovirus‐treated myotubes (*n* = 3). Each point represents an individual experiment. *p < 0.05, **p < 0.01 and ***p < 0.001. ns, no significant differences were observed.
**Figure S6: There were no significant changes in PKA, AKT activity and PP2A expression levels in the muscles of KAL‐TG mice.** (**a**) Representative western blots and quantification of p‐PKA/PKA, p‐AKT/AKT and PP2A in 3‐month‐old WT and KAL‐TG mice (*n* = 3). ns, no significant differences were observed.
**Figure 7. KAL‐mediated AMPK inhibition is neither dependent on LRP6 nor associated with inhibition of adiponectin/AdipoR1/PPL1 expression.** (**a**) Representative immunoblotting and quantitative analysis of nonphosphorylated β‐catenin (non‐p‐β‐catenin) and total β‐catenin levels in muscle of 3‐month‐old WT and KAL‐TG mice. (**b**) Representative immunoblot analysis of p‐AMPK/AMPK levels in C2C12 myotubes overexpressing KAL following LRP6 knockdown (si‐LRP6) (*n* = 3). Each point represents an individual experiment. (**c**) Comparison of transcription levels of AdipoR1 and LRP6 in gastrocnemius (*n* = 6). (**d**) Predicted binding affinity of KAL with AdipoR1 and LRP6. (**e**) AdipoR1 mRNA expression levels in gastrocnemius of 3‐month‐old mice (*n* = 6). (**f**) Representative immunoblotting and densitometric analysis of AdipoR1 protein levels in gastrocnemius of 3‐month‐old mice (*n* = 6). (**g**) Representative immunoblots and statistical analysis of APPL1 and adiponectin in skeletal muscle of 3‐month‐old mice (*n* = 3). *p < 0.05 and ***p < 0.001. ns, no significant differences were observed.
