# Supplementary material for: Elevated Kallistatin Induces Myosteatosis and Exercise Intolerance by Antagonizing AdipoR1‐Mediated AMPK Signalling
Source: J Cachexia Sarcopenia Muscle. 2026 Apr 1;17(2):e70261. doi: 10.1002/jcsm.70261 (PMC13045455; doi:10.1002/jcsm.70261)
Supplement: Supplementary file 1 — Data S1: Supplementary information. [file JCSM-17-e70261-s001.pdf]

60 kcal%脂肪热量高脂鼠粮

(High Fat (60 FDC) Purified Rodent Diet)

货号：HF60 (美国货号 112252)

储存条件：-20 度，保持干燥

Ingredient，成分	单位热量值（kcal/g）	重量（gram）	热量（kcal）
Caisein（酪蛋白）	4	200	800
L-Cystine（L-胱氨酸）	4	3	12
Sucrose（蔗糖）	4	68.8	275
Dyetrose（麦芽糊精）	4	125	500
Lard（猪油）	9	245	2205
Soybean Oil（大豆油，含 TBHQ）	9	25	225
Cellulose（纤维素）	0	50	0
Mineral Mix #210088（复合矿物质）	1.6	10	16
Calcium Carbonate（碳酸钙）	0	5.5	0
Dicalcium Phosphate（磷酸氢钙）	0	13	0
Potassium Citrate H2O（1 水柠檬酸钾）	0	16.5	0
Vitamin Mix # 300050（复合维生素）	3.9	10	39
Choline Bitartrate（酒石酸胆碱）	0	2	0
Blue Dye（蓝色色素）	0	0.05	0
	合计	**773.85**	**4072**

	gm%（质量比）	kcal%（热量比）
Protein（蛋白）	26	20
Carbohydrate（碳水化合物）	26	20
Fat（脂肪）	35	60
kcal/gm	5.26	

引用文献：

1. Xia, Y., Lan, J., Yang, J. *et al.* Saturated fatty acid-induced neutrophil extracellular traps contribute to exacerbation and biologic therapy resistance in obesity-related psoriasis. *Cell Mol Immunol* **22**, 597–611 (**2025**). https://doi.org/10.1038/s41423-025-01278-7

戴茨生物科技（无锡）有限公司
地址：安徽省滁州市凤阳县刘府镇刘蚌路戴茨科技园
电话：139 2528 4779
info@dyets-cn.com
www.dyets-cn.com

2. Wei S, Shou D, Huang S. *et al*. Nonalcoholic Fatty Liver Disease Exacerbates the Advancement of Renal Fibrosis by Modulating Renal CCR2+PIRB+ Macrophages Through the ANGPTL8/PIRB/ALOX5AP Axis. Adv Sci (Weinh). **2025** Dec;12(46):e09351. doi: 10.1002/advs.202509351.
3. Jiang S, Jia H. *et al*. Multimodal analysis stratifies genetic susceptibility and reveals the pathogenic mechanism of kidney injury in diabetic nephropathy. Cell Rep Med. **2025** Aug 19;6(8):102249.
4. Deng, Z., Zhao, L., Li, S. *et al.* Targeting dysregulated phago-/auto-lysosomes in Sertoli cells to ameliorate late-onset hypogonadism. *Nat Aging* **4**, 647–663 (**2024**).
5. Meiling Yan, *et al.* USP7 promotes cardiometabolic disorders and mitochondrial homeostasis dysfunction in diabetic mice via stabilizing PGC1β. Pharmacological Research,Volume 205, **2024**, 107235
6. Zhan-Ming Li, *et al*. Host ALDH2 deficiency aggravates nonalcoholic steatohepatitis through gut-liver axis,Pharmacological Research,Volume 196, **2023**, 106902
7. Yan Yin, *et al*. Preventive Effects of Apple Polyphenol Extract on High-Fat-Diet-Induced Hepatic Steatosis Are Related to the Regulation of Hepatic Lipid Metabolism, Autophagy, and Gut Microbiota in Aged Mice. Journal of Agricultural and Food Chemistry **2023** 71 (50), 20011-20033
8. Cui Y, *et al*. Apple polyphenol extract modulates bile acid metabolism and gut microbiota by regulating the circadian rhythms in daytime-restricted high fat diet feeding C57BL/6 male mice. Food Funct. **2022** Mar 7;13(5):2805-2822.
9. RJ Perry, *et al*. Leptin's hunger-suppressing effects are mediated by the hypothalamic–pituitary–adrenocortical axis in rodents. PNAS July 2, **2019** 116 (27) 13670-13679
10. CL. Miranda *et al.* Non-estrogenic Xanthohumol Derivatives Mitigate Insulin Resistance and Cognitive Impairment in High-Fat Diet-induced Obese Mice.Sci Rep. **2018** Jan 12;8(1):613.
11. H Coia，et al. Prevention of Lipid Peroxidation–derived Cyclic DNA Adduct and Mutation in High-Fat Diet–induced Hepatocarcinogenesis by Theaphenon E. Cancer Prev Res; 11(10) October **2018**
12. SJ Mitchell *et al.* Nicotinamide Improves Aspects of Healthspan, but Not Lifespan, in Mice. Cell Metabolism。**2018**, 27, 667–676
13. J Guo *et al.* High-dose metformin (420 mg/kg daily po) increases insulin sensitivity but does not affect neointimal thickness in the rat carotid balloon injury model of restenosis. METABOLISM CLINICAL AND EXPERIMENTAL 68 (**2017**) 108 – 118
14. AK Kopec，et al. Thrombin promotes diet-induced obesity through fibrin-driven inflammation. The Journal of Clinical Investigation. **2017** Aug;127(8):3152-3166.
15. GH Norris *et al.* Dietary sphingomyelin attenuates hepatic steatosis and adipose tissue inflammation in high-fat-diet-induced obese mice. J Nutr Biochem. **2017** Feb;40:36-43.
16. JW Yang, et al. GPR119: a promising target for nonalcoholic fatty liver disease. FASEB J. 30, 324–335 (2016).
17. MM Kotani, et al. Pivotal role of liver sinusoidal endothelial cells in NAFLD/NASH progression. Lab Invest 95, 1130–1144 (**2015**)
18. MG Tordoff，et al. No effects of monosodium glutamate consumption on the body weight or composition of adult rats and mice. Physiology & Behavior 107 (**2012**) 338–345
19. YJ Lee, et al. Nuclear receptor PPARγ-regulated monoacylglycerol O-acyltransferase 1 (MGAT1) expression is responsible for the lipid accumulation in diet-induced hepatic steatosis. PNAS August 21, **2012** 109 (34) 13656-13661
20. SH Hwahng, et al. Role of Adenosine Monophosphate-Activated Protein Kinase–p70 Ribosomal S6 Kinase-1 Pathway in Repression of Liver X Receptor-Alpha–Dependent Lipogenic Gene Induction and Hepatic Steatosis by a Novel Class of Dithiolethiones. HEPATOLOGY **2009**;49:1913-1925
