# Supplementary material for: Elevated Kallistatin Induces Myosteatosis and Exercise Intolerance by Antagonizing AdipoR1‐Mediated AMPK Signalling
Source: J Cachexia Sarcopenia Muscle. 2026 Apr 1;17(2):e70261. doi: 10.1002/jcsm.70261 (PMC13045455; doi:10.1002/jcsm.70261)
Supplement: Supplementary file 2 — Table S1: Interference sequence and primer sequence. Table S2: Antibodies information. Table S3: Reagent information. Table S3: Triglyceride content in the gastrocnemius muscle of 3‐month‐old mice. Table S4: Triglyceride content in the gastrocnemius muscle of 6‐month‐old mice. Table S5: Triglyceride content in the gastrocnemius muscle of 9‐month‐old mice. Table S6: Triglyceride content in the gastrocnemius muscle of 13‐month‐old mice. Table S7: Triglyceride content in the gastrocnemius muscle of rats after control and high‐fat diet. Table S8: Triglyceride content in the gastrocnemius muscle of rats after control and high‐fructose intake. Table S9: Triglyceride content in the gastrocnemius muscle of rats after AdipoRon and fenofibrate treatment. Table S10: The running distance of 3‐month‐old mice during exercise endurance experiments. Table S11: The running distance of 6‐month‐old mice during exercise endurance experiments. Table S12: The running distance of 9‐month‐old mice during exercise endurance experiments. Table S13: The running distance of mice during exercise endurance experiments after AdipoRon and fenofibrate treatment. [file JCSM-17-e70261-s003.docx]

**Elevated Kallistatin induces myosteatosis and exercise intolerance by antagonizing AdipoR1-mediated AMPK signaling**

**Fuyan Hong^1,3#^, Zhenzhen Fang^1#^, Gang Shen^6#^, Yan Wang^1^, Yunhua Li^1^, Youbin Zhong^1^, Junze Dai^1^, Chengwei Zhang^1^, Jing Zhang^1^, Wencan Chen^1^, Weiwei Qi^1^, Xia Yang^1,4^, Guoquan Gao^1,4^*, Ti Zhou^1,2,5^***

^1^Department of Biochemistry and Molecular Biology, Zhongshan School of Medicine, Sun Yat-sen University, Guangzhou, 510080, China.

^2^ China Key Laboratory of Tropical Disease Control (Sun Yat-sen University), Ministry of Education; Guangzhou, 510080, China.

^3^ Department of Laboratory Medicine, The Second Affiliated Hospital of Guangzhou University of Chinese Medicine, Guangzhou 510120, China.

^4^Guangdong Engineering & Technology Research Center for Gene Manipulation and Bio-macromolecular Products, Sun Yat-Sen University; Guangzhou, 510080, China.

^5^ Guangdong Provincial Key Laboratory of Diabetology, Guangzhou, Guangdong, China.

^6^ Department of Laboratory Medicine, Third Affiliated Hospital of Sun Yat-Sen University, Tianhe Road 600#, Guangzhou 510630, China.

**Contact**: Prof. Ti Zhou: Email: zhouti2@mail.sysu.edu.cn; Address: Department of Biochemistry and Molecular Biology, Zhongshan School of Medicine, Sun Yat-sen University, 74 Zhongshan 2nd Road, Guangzhou 510080, China.

*Co-corresponding authors.

# Contributed equally.

**Supplementary materials and methods**

**Cell Transfection**

Adenoviruses overexpressing KAL with red fluorescence and C-segment Flag tags (Ad-KAL) and adenoviruses overexpressing red fluorescence protein as controls (Ad-con) were purchased from Bio Technology Corp Ltd (Shanghai, China). The C2C12 cells were treated with adenovirus on the fourth day of differentiation, and samples were collected 48h later. AdipoR1 siRNA was purchased from Tsingke Biotechnology. AdipoR1(si-AdipoR1) or control (si-CON) siRNA was transfected using lipo3000 (Thermo fisher). At least 3 biological replicates were conducted for each cell experiment. The Interference sequence is listed in Supplementary Table 1.

**ELISA**

KAL levels in serum and cell supernatants were measured using an ELISA kit (DY1669) from R&D Systems.

**Histological Analyses**

For Oil Red O staining, 10μm tissue sections were used. After the frozen sections were brought to room temperature, they were gently immersed in Oil Red O working solution and incubated in the dark for 8-10 minutes. The sections were then briefly immersed in two changes of 60% isopropanol for 3-5 seconds each, followed by two rinses in pure water for 10 seconds each. Finally, the nuclei were counterstained with hematoxylin.

**Immunofluorescence Staining**

After deparaffinization and rehydration through graded ethanol concentrations, 5μm-thick tissue sections were stained with primary antibodies overnight at 4℃. The sections were then rinsed with PBS and incubated with fluorochrome-conjugated secondary antibodies (1:100 dilution) for 1 hour at room temperature. Nuclei were stained with DAPI (1μg/ml) after washing with PBS. Images were captured using the AxioScan.Z1 (Zeiss), an automated digital slide scanning system.

**RNA Isolation and RT-qPCR**

RNA was extracted using a commercial kit (CWBIO, CW0581, China), and reverse transcription was performed using the Accurate Biology reverse transcription reagent kit (AG11706, China). RT-qPCR was performed using SYBR® Green I (Abclonal, China) on a real-time fluorescent quantitative PCR system (Bio-Rad, USA). Primer sequences are listed in Supplementary Table 1.

**Immunoblotting Analysis**

Muscle or cellular protein lysates were quantified using a BCA assay (KeyGen BioTECH). Equal amounts of protein were separated by SDS-PAGE and transferred to PVDF membranes (Millipore). Membranes were incubated overnight at 4°C with primary antibodies, followed by species-matched HRP-conjugated secondary antibodies. After TBST washes, proteins were detected by enhanced chemiluminescence (Bio-Rad). Antibody specifications are provided in Supplementary Table 2.

**Co-immunoprecipitation (Co-IP) Assay**

HEK293T cells or differentiated C2C12 cells were transfected with an adenovirus expressing Kallistatin-Flag for 48 hours and then lysed for immunoprecipitation. After quantification using the BCA protein assay, cell lysates were divided into the input and IP groups and subjected to immunoblotting.

**Detection of Cellular ATP Levels**

Intracellular ATP levels were measured according to the manufacturer’s instructions (Beyotime Biotechnology, S0026). Briefly, differentiated C2C12 cells or muscle tissues were homogenized in lysis buffer and mixed with ATP reagent containing luciferase. ATP levels were quantified using chemiluminescence (TECAN, Infinite F500). Protein concentrations were determined using the BCA protein assay for normalization.

**Detection of Cellular Ca^2+^ Levels**

Fluo-4, AM (YEASEN, 40704ES72) was used for the detection of intracellular Ca^2+^ concentration. Differentiated C2C12 cells were incubated with HBSS containing 4 μM Fluo-4, AM at 37 °C for 60 min, followed by three washes with HBSS. Fluorescence intensity was measured using a multi-mode analyzer (Victor Nivo 5S) after incubating with fresh HBSS for an additional 30 minutes.

**Mito Tracker Staining**

The cell culture supernatant was removed, and the cells were gently washed three times with PBS. Cells were fixed with 4% paraformaldehyde at room temperature for 15 minutes, followed by permeabilization with 0.2% Triton-X100 in PBS for 10 minutes. After washing, the cells were incubated with a 2 μM BODIPY 493/503 working solution in the dark for 20 minutes. Cells were then washed three times with PBS and imaged using an inverted fluorescence microscope.

**Treadmill Running Assay**

The treadmill running assay was performed as described in the previous literature [1]. Mice were acclimated to the treadmill at a low speed (5 m/min) for three consecutive days, with the treadmill kept level and without incline, for 5 minutes each day. On the fourth day, the running test was conducted on a treadmill set at a 5° incline. The animals ran at 10 m/min for the first 10 minutes, with the speed increasing by 2 m/min every 5 minutes until exhaustion. Exhaustion was defined as the inability to continue running for more than 5 seconds. The total distance run and duration were recorded.

**Indirect Calorimetry Experiments**

Indirect calorimetry was performed on 6-month-old WT and KAL-TG mice using a Promethion HD Multiplexed Respirometry System (Sable Systems). Oxygen consumption rate (VO2) and carbon dioxide production rate (VCO2) were measured at 1-second intervals, with cages sampled for 30 seconds every 5 minutes. Energy expenditure was calculated using the Weir equation (Energy expenditure = 3.941 kcal/L × VO2 + 1.106 kcal/L × VCO2). Energy expenditure was calculated using the Weir equation (Energy expenditure = 3.941 kcal/L × VO2 + 1.106 kcal/L × VCO2). Data were analyzed using CaIR ANCOVA analysis (https://calrapp.org/) for regression analysis of energy expenditure in mice [2].

**References for the Supplementary Methods section**

1. Knudsen, N.H., K.J. Stanya, A.L. Hyde, et al., Interleukin-13 drives metabolic conditioning of muscle to endurance exercise. Science, 2020. 368(6490).

2. Mina, A.I., R.A. LeClair, K.B. LeClair, et al., CalR: A Web-Based Analysis Tool for Indirect Calorimetry Experiments. Cell Metab, 2018. 28(4): p. 656-666 e1.

**Supplementary References**

S1. Chen, L. L.; Zhang, H. H.; Zheng, J.; Hu, X.; Kong, W.; Hu, D., et al., Resveratrol attenuates high-fat diet-induced insulin resistance by influencing skeletal muscle lipid transport and subsarcolemmal mitochondrial β-oxidation. Metabolism: clinical and experimental 2011, 60 (11), 1598-609.

S2. Rai, A. K.; Jaiswal, N.; Maurya, C. K.; Sharma, A.; Ahmad, I.; Ahmad, S., et al., Fructose-induced AGEs-RAGE signaling in skeletal muscle contributes to impairment of glucose homeostasis. The Journal of nutritional biochemistry 2019, 71, 35-44.

S3. Yan, Y.; Tao, H.; He, J.; Huang, S. Y., The HDOCK server for integrated protein-protein docking. Nature protocols 2020, 15 (5), 1829-1852.

S4. Yugandhar, K.; Gromiha, M. M., Protein-protein binding affinity prediction from amino acid sequence. Bioinformatics (Oxford, England) 2014, 30 (24), 3583-9.

S5. Kadowaki, T.; Yamauchi, T., Adiponectin and adiponectin receptors. Endocrine reviews 2005, 26 (3), 439-51.

S6. Boutari, C.; Mantzoros, C. S., Adiponectin and leptin in the diagnosis and therapy of NAFLD. Metabolism: clinical and experimental 2020, 103, 154028.

S7. Mantovani, A.; Zusi, C.; Csermely, A.; Salvagno, G. L.; Colecchia, A.; Lippi, G., et al., Association between lower plasma adiponectin levels and higher liver stiffness in type 2 diabetic individuals with nonalcoholic fatty liver disease: an observational cross-sectional study. Hormones (Athens, Greece) 2022, 21 (3), 477-486.

S8. Liu, M.; Lim, S. T.; Song, W.; Coffman, T. M.; Wang, X., Beyond lipids: fenofibrate in diabetic retinopathy and nephropathy. Trends in pharmacological sciences 2025.

S9. Nieuwdorp, M.; Stroes, E. S.; Kastelein, J. J., Normalization of metabolic syndrome using fenofibrate, metformin or their combination. Diabetes, obesity & metabolism 2007, 9 (6), 869-78.

**Supplementary Tables**

**Supplementary Table 1. Interference sequence and primer sequence.**

| **Name** | **Sequence（5’ to 3’）** |
| --- | --- |
| **h-*SERPINA4*-F** | GCATCTTCCCAAGTTCTCCATT |
| **h-*SERPINA4*-R** | ATGCCGGATAAGTCAGCCCA |
| **Rat-*Serpina4*-F** | AGGAGGAATTGTTCCATCTGAGAG |
| **Rat-*Serpina4*-R** | GGTCTGGCTGACTGTGAGAG |
| **m-*Actb*-F** | GGCTGTATTCCCCTCCATCG |
| **m-*Actb*-R** | CCAGTTGGTAACAATGCCATGT |
| **Rat-*Actb*-F** | GAGAGGGAAATCGTGCGTGA |
| **Rat-*Actb*-R** | CAGGGAGGAAGAGGATGCGG |
| **m-*Hexo*-F** | GCCAGCCTCTCCTGATTTTAGTGT |
| **m-*Hexo*-R** | GGGAACACAAAAGACCTCTTCTGG |
| **m-*16sRNA*-F** | CCGCAAGGGAAAGATGAAAGAC |
| **m-*16sRNA*-R** | TCGTTTGGTTTCGGGGTTTC |
| **m- *Ppargc1a*-F** | AGACGGATTGCCCTCATTTGA |
| **m- *Ppargc1a* -R** | GGTCTTAACAATGGCAGGGTTT |
| **m- *Nrf1*-F** | GGCTGATGGAGAGGTGGAAC |
| **m- *Nrf1* -R** | GGCTTCTGCCAGTGATGCTA |
| **m- *Nrf2*-F** | GACTACAGTCCCAGCAGAGTGA |
| **m- *Nrf2*-R** | GTGCTCAGAAACCTCCTTCCA |
| **m- *Tfam*-F** | GAATGTGGAGCGTGCTAAAAG |
| **m- *Tfam* -R** | TCGGAATACAGACAAGACTGATAG |
| **m-*Adipor1*-F** | AGACAACGACTACCTGCTACA |
| **m-*Adipor1*-R** | GTGGATGCGGAAGATGCTCT |
| **m-*Lrp6*-F** | TGCAAACAGACGGGACTTGAG |
| **m-*Lrp6*-R** | CGGGGACAATAATCCAGAAACAA |
| **m-si-*AdipoR1*** | AUAUUUGGUCUCAGCAUCGUC |

**Supplementary Table 2. Antibodies information.**

| **Antibodies** | **Supplier** | **Catalog number** | **dilution** |
| --- | --- | --- | --- |
| **Kallistatin** | Abcam | ab187656 | 1:1000 |
| **CGI-58** | Santa Cruz | sc-100468 | 1:200 |
| **TOM20** | ZENBIO | 381087 | 1:1000 |
| **Oxphos** | invitrogen | 45-8199 | 1:2000 |
| **Parkin** | WanleiBio | WL02512 | 1:1000 |
| **LC3B** | ABclonal | A7198 | 1:1000 |
| **ATGL** | cayman | 10006409 | 1:1000 |
| **CPT1β** | ABclonal | A6796 | 1:1000 |
| **FASN** | CST | 3180 | 1:1000 |
| **CD36** | Abcam | ab133625 | 1:1000 |
| **FATP4** | Santa cruz | Sc-393309 | 1:100 |
| **HSL** | CST | 18381S | 1:1000 |
| **p-HSL** | CST | 4126S | 1:1000 |
| **ACC** | CST | 3676 | 1:1000 |
| **p-ACC** | CST | 3661 | 1:1000 |
| **PGC1α** | ZENBIO | 381615 | 1:1000 |
| **NRF1** | ABclonal | A5547 | 1:1000 |
| **β-actin** | Sigma | A5441 | 1:5000 |
| **GAPDH** | Sigma | G8795 | 1:5000 |
| **Anti-rabbit** | Vector Laboratory | PI-1000 | 1:1000 |
| **Anti-mouse** | Vector Laboratory | PI-2000 | 1:1000 |
| **p-AMPK** | CST | 4188S | 1:1000 |
| **AMPK** | CST | 2532S | 1:1000 |
| **p-LKB1** | Santa cruz | Sc-271924 | 1:100 |
| **LKB1** | Santa cruz | Sc-32245 | 1:100 |
| **CaMKK2** | Santa cruz | Sc-271674 | 1:200 |
| **p-PKA** | CST | 5661S | 1:1000 |
| **PKA** | Proteintech | 67491 | 1:1000 |
| **p-AKT** | CST | 4060S | 1:1000 |
| **AKT** | CST | 4691S | 1:1000 |
| **PP2A** | ZENBIO | 251610 | 1:1000 |
| **Non-p-β-catenin** | CST | 8814S | 1:1000 |
| **β-catenin** | CST | 9562S | 1:1000 |
| **LRP6** | CST | 3395S | 1:1000 |
| **AdipoR1** | Santa cruz | 518030 | 1:200 |
| **APPL1** | Santa cruz | Sc-271901 | 1:200 |
| **Adiponectin** | ABclonal | A2543 | 1:1000 |
| **LKB1** | Abclonal | A2122 | 1:1000 |
| **AdipoR1** | Abclonal | A21533 | 1:1000 |
| **APPL1** | PTM BIO | PTM-20123 | 1:1000 |

**Supplementary Table 3. Reagent information.**

| **Reagent name** | **Supplier** | **Catalog number** |
| --- | --- | --- |
| **AICAR** | Selleck | 2627-69-2 |
| **AdipoRon** | Selleck | 924416-43-3 |
| **Recombinant human kallistatin protein** | MCE | HY-P71143 |
| **Fenofibrate** | Sigma-Aldrich | 49562-28-9 |

**Supplementary Table 3. Triglyceride content in the gastrocnemius muscle of 3-month-old mice**

| Experimental groups | Mean ± SD (μmol/g) | 95% CI limits | | P-value  (vs. Control) |
| --- | --- | --- | --- | --- |
|  |  | Lower | Upper |  |
| WT | 64.65 ± 18.95 | 44.76 | 84.53 | - |
| KAL-TG | 61.18 ± 7.724 | 53.07 | 69.28 | 0.6870 |

**Supplementary Table 4. Triglyceride content in the gastrocnemius muscle of 6-month-old mice**

| Experimental groups | Mean ± SD (μmol/g) | 95% CI limits | | P-value  (vs. Control) |
| --- | --- | --- | --- | --- |
|  |  | Lower | Upper |  |
| WT | 54.26 ± 14.56 | 38.99 | 69.54 | - |
| KAL-TG | 85.13 ± 11.53 | 73.03 | 97.24 | 0.0022 |

**Supplementary Table 5. Triglyceride content in the gastrocnemius muscle of 9-month-old mice**

| Experimental groups | Mean ± SD (μmol/g) | 95% CI limits | | P-value  (vs. Control) |
| --- | --- | --- | --- | --- |
|  |  | Lower | Upper |  |
| WT | 81.11 ± 19.50 | 60.65 | 101.6 | - |
| KAL-TG | 136.8 ± 48.55 | 85.85 | 187.7 | 0.0262 |

**Supplementary Table 6. Triglyceride content in the gastrocnemius muscle of 13-month-old mice**

| Experimental groups | Mean ± SD (μmol/g) | 95% CI limits | | P-value  (vs. Control) |
| --- | --- | --- | --- | --- |
|  |  | Lower | Upper |  |
| WT | 88.04 ± 18.33 | 42.50 | 133.6 | - |
| KAL-TG | 267.1 ± 40.04 | 167.7 | 366.6 | 0.0021 |

**Supplementary Table 7. Triglyceride content in the gastrocnemius muscle of rats after control and high-fat diet**

| Experimental groups | Mean ± SD (μmol/g) | 95% CI limits | | P-value |
| --- | --- | --- | --- | --- |
|  |  | Lower | Upper |  |
| WT+CON | 68.17 ± 13.88 | 53.60 | 82.73 | - |
| WT+HFD | 136.4 ± 16.68 | 118.9 | 153.9 | 0.0002 (vs. WT+CON) |
| *SERPINA4^-/-^*+CON | 82.45 ± 28.11 | 52.95 | 111.9 | 0.0020 (vs. WT+HFD) |
| *SERPINA4^-/-^*+HFD | 81.91 ± 25.59 | 55.06 | 108.8 | 0.0018 (vs. WT+HFD) |

**Supplementary Table 8. Triglyceride content in the gastrocnemius muscle of rats after control and high-fructose intake**

| Experimental groups | Mean ± SD (μmol/g) | 95% CI limits | | P-value |
| --- | --- | --- | --- | --- |
|  |  | Lower | Upper |  |
| WT+CON | 101.2 ± 12.54 | 85.58 | 116.7 | - |
| WT+HFru | 152.6 ± 26.93 | 119.2 | 186.1 | 0.0050 (vs. WT+CON) |
| *SERPINA4^-/-^*+CON | 101.7 ± 23.96 | 73.05 | 126.0 | 0.0039 (vs. WT+HFru) |
| *SERPINA4^-/-^*+HFru | 107.6 ± 17.72 | 85.65 | 129.6 | 0.0141 (vs. WT+HFru) |

**Supplementary Table 9. Triglyceride content in the gastrocnemius muscle of rats after AdipoRon and Fenofibrate treatment**

| Experimental groups | Mean ± SD (μmol/g) | 95% CI limits | | P-value |
| --- | --- | --- | --- | --- |
|  |  | Lower | Upper |  |
| WT+CON | 52.22 ± 7.282 | 43.17 | 61.26 | - |
| KAL-TG+CON | 106.7 ± 27.88 | 72.06 | 141.3 | 0.0107 (vs. WT+CON) |
| KAL-TG+Adi | 59.55 ± 24.13 | 29.60 | 89.51 | 0.0287 (vs. KAL-TG+CON) |
| KAL-TG+Feno | 67.79 ± 28.83 | 31.99 | 103.6 | 0.0823 (vs. KAL-TG+CON) |

Supplementary Table 10. The running distance of 3-month-old mice during exercise endurance experiments

| Experimental groups | Mean ± SD (m) | 95% CI limits | | P-value  (vs. Control) |
| --- | --- | --- | --- | --- |
|  |  | Lower | Upper |  |
| WT | 230.7 ± 57.81 | 159.0 | 302.5 | - |
| KAL-TG | 252.6 ± 68.55 | 167.4 | 337.7 | 0.6012 |

Supplementary Table 11. The running distance of 6-month-old mice during exercise endurance experiments

| Experimental groups | Mean ± SD (m) | 95% CI limits | | P-value |
| --- | --- | --- | --- | --- |
|  |  | Lower | Upper |  |
| WT | 186.8 ± 36.18 | 156.6 | 217.1 | - |
| KAL-TG | 154.9 ± 20.64 | 137.7 | 172.2 | 0.0480 |

Supplementary Table 12. The running distance of 9-month-old mice during exercise endurance experiments

| Experimental groups | Mean ± SD (m) | 95% CI limits | | P-value |
| --- | --- | --- | --- | --- |
|  |  | Lower | Upper |  |
| WT | 179.5 ± 49.92 | 127.1 | 231.9 | - |
| KAL-TG | 112.3 ± 32.51 | 78.22 | 146.5 | 0.0200 |

Supplementary Table 13. The running distance of mice during exercise endurance experiments after AdipoRon and Fenofibrate treatment

| Experimental groups | Mean ± SD (m) | 95% CI limits | | P-value |
| --- | --- | --- | --- | --- |
|  |  | Lower | Upper |  |
| WT+con | 298.9 ± 32.50 | 258.6 | 339.3 | - |
| KAL-TG+con | 190.2 ± 48.89 | 129.5 | 250.9 | 0.0080 (vs. WT+con) |
| KAL-TG+Adi | 280.4 ± 53.12 | 214.5 | 346.4 | 0.0291 (vs. KAL-TG+con) |
| KAL-TG+Feno | 292.7 ± 44.72 | 237.1 | 348.2 | 0.0124 (vs. KAL-TG+con ) |

**Supplementary Figure legends**

**Supplement Figure 1.** **There is no significant difference in body weight and muscle weight between KAL-TG and WT mice.** (**a-b**) *SERPINA4* mRNA levels in human liver tissues from GEO datasets GSE89632 (**a**) and GSE23343 (**b**). (**c**) Genotyping results of WT and KAL-TG mice. (d-g) Body weight and muscle mass measurements in KAL-TG and WT mice at 3 months (d), 6 months (e), 9 months (f), and 13 months (g) of age. 3-, 6-, and 9-month-old groups, *n* = 6 each; 13-month-old group, *n* = 3. **p* < 0.05, ***p* < 0.01. ns, no significant differences were observed.

**Supplement Figure 2. Changes in muscle lipids of KAL-TG mice.** (**a-d**) Muscle cholesterol (CHO) and fatty acid (FA) content in 3-month-old (**a**), 6-month-old (**b**), 9-month-old (**c**), and 13-month-old (**d**) mice. 3-, 6-, and 9-month-old groups, *n* = 6 each; 13-month-old group, *n* = 3. (e-h) The levels of serum total cholesterol (TC), HDL-C, LDL-C, total triglycerides (TG), and free fatty acids (FFA) in mice at 3-month-old (e, *n* of WT = 4, *n* of KAL-TG = 5), 6-month-old (f, *n* of WT = 9, *n* of KAL-TG = 8), 9-month-old (g, *n* = 8), and 13-month-old (h, *n* of WT = 5, *n* of KAL-TG = 7) mice. (i) ATP levels in the gastrocnemius from mice at different ages. 3-, 6-, and 9-month-old groups, *n* = 6 each; 13-month-old group, *n* = 3. (j) Chronological progression of phenotypic manifestations in KAL-TG mice. (k) qPCR results of hepatic *Serpina4* (*n* = 5). **p* < 0.05, ***p* < 0.01. ns, no significant differences were observed.

**Supplement Figure 3. Pathological upregulation of KAL promotes aberrant de novo lipogenesis through AMPK.** (**a**) KAL levels in the culture supernatant of adenovirus-treated myotubes (*n* = 3). (**b**) Relative fatty acid uptake in myotubes following adenovirus treatment (*n* = 5). (**c-d**) Representative immunoblot of lipid metabolism regulators in the gastrocnemius of 6-month-old mice: (**c**) CD36, FATP4, and p-HSL/HSL; (**d**) FASN, ATGL, and CGI58. (**e**) Representative immunoblots and quantification of p-ACC/ACC and FASN in the gastrocnemius of 13-month-old mice. (**f**) Representative Oil Red O and BODIPY staining in adenovirus-treated myotubes. Scale bars, 100 μm. (**g**) Representative western blot and quantification of p-ACC/ACC in gastrocnemius muscle from high-fat diet-fed mice. (**h**) Representative immunoblotting and statistical analysis of p-AMPK/AMPK in rat gastrocnemius muscle after high-fat diet and high-fructose intake. ***p* < 0.01, ****p* < 0.001 or *****p* < 0.0001. ns, no significant differences were observed.

**Supplementary Figure 4. Pathologically elevated KAL reduces mitochondrial content.** (**a**) Mitochondrial DNA copy number in the gastrocnemius of 3-month-old mice (*n* = 3). (**b**) Representative immunoblot of oxidative phosphorylation (OXPHOS)-related protein and TOM20 levels in the gastrocnemius of 3-month-old mice and quantitative analysis. (**c**) Food intake in 6-month-old mice (*n* = 4). (**d**) Representative immunoblot and quantitative analysis of OXPHOS-related protein levels in the gastrocnemius muscle of 13-month-old mice. (**e**) ATP levels in adenovirus-treated differentiated myotubes (*n* = 3). (**f**) Representative Mitotracker staining of mitochondria in myotubes treated with adenovirus for 48h or 0.5 μg/mL rhKAL for 24h. Scale bars, 100 μm. (**g**) Representative immunoblot and quantification of CPT1β, OXPHOS-related proteins, and TOM20 in gastrocnemius from rats after high-fat diet or high-fructose intake. **p* < 0.05, ***p* < 0.01, ****p* < 0.001. ns, no significant differences were observed.

**Supplemental Figure 5. KAL regulates mitochondrial biogenesis.** (**a**) mRNA expression levels of *Ppargc1α*, *Tfam*, *Nrf1*, and *Nrf2* in the gastrocnemius muscle of 3-month-old mice (*n* = 4). (**b**) Representative immunoblot analysis of PGC1α and NRF1 in myotubes transduced with adenovirus and corresponding quantification (*n* = 3). Each point represents an individual experiment. (**c**) Representative immunoblots and quantification analysis of PGC1α and NRF1 in myotubes treated with rhKAL protein (*n* = 3). Each point represents an individual experiment. (**d**) LC3B-II/LC3B-I ratio in the gastrocnemius of 6-month-old mice, assessed by immunoblotting and quantified. (**e**) Representative immunoblots and statistical analysis of Parkin and LC3B-II/LC3B-I in cytoplasmic and mitochondrial fractions of adenovirus-treated myotubes (*n* = 3). Each point represents an individual experiment. **p* < 0.05, ***p* < 0.01, ****p* < 0.001. ns, no significant differences were observed.

**Supplement Figure 6. There were no significant changes in PKA, AKT activity, and PP2A expression levels in the muscles of KAL-TG mice.** (**a**) Representative western blots and quantification of p-PKA/PKA, p-AKT/AKT, and PP2A in 3-month-old WT and KAL-TG mice (*n* = 3). ns, no significant differences were observed.

**Supplement Figure 7. KAL-mediated AMPK inhibition is neither dependent on LRP6 nor associated with inhibition of Adiponectin/AdipoR1/PPL1 expression.** (**a**) Representative immunoblotting and quantitative analysis of non-phosphorylated β-catenin (non-p-β-catenin) and total β-catenin levels in muscle of 3-month-old WT and KAL-TG mice. (**b**) Representative immunoblot analysis of p-AMPK/AMPK levels in C2C12 myotubes overexpressing KAL following LRP6 knockdown (si-LRP6) (*n* = 3). Each point represents an individual experiment. (**c**) Comparison of transcription levels of AdipoR1 and LRP6 in gastrocnemius (*n* = 6). (**d**) Predicted binding affinity of KAL with AdipoR1 and LRP6. (**e**) AdipoR1 mRNA expression levels in gastrocnemius of 3-month-old mice (*n* = 6). (**f**) Representative immunoblotting and densitometric analysis of AdipoR1 protein levels in gastrocnemius of 3-month-old mice (*n* = 6). (**g**) Representative immunoblots and statistical analysis of APPL1 and Adiponectin in skeletal muscle of 3-month-old mice (*n* = 3). **p* < 0.05, ****p* < 0.001. ns, no significant differences were observed.
