# Supplementary material for: Elevated Kallistatin Induces Myosteatosis and Exercise Intolerance by Antagonizing AdipoR1‐Mediated AMPK Signalling
Source: J Cachexia Sarcopenia Muscle. 2026 Apr 1;17(2):e70261. doi: 10.1002/jcsm.70261 (PMC13045455; doi:10.1002/jcsm.70261)
Supplement: Supplementary file 3 — Figure S1: jcsm70261‐sup‐0003‐Supplementary_Figures.pdf. There is no significant difference in body weight and muscle weight between KAL‐TG and WT mice. (a–b) SERPINA4 mRNA levels in human liver tissues from GEO datasets GSE89632 (a) and GSE23343 (b). (c) Genotyping results of WT and KAL‐TG mice. (d–g) Body weight and muscle mass measurements in KAL‐TG and WT mice at 3 months (d), 6 months (e), 9 months (f) and 13 months (g) of age. 3‐, 6‐ and 9‐month‐old groups, n = 6 each; 13‐month‐old group, n = 3. *p < 0.05 and **p < 0.01. ns, no significant differences were observed. Figure S2: Changes in muscle lipids of KAL‐TG mice. (a–d) Muscle cholesterol (CHO) and fatty acid (FA) content in 3‐month‐old (a), 6‐month‐old (b), 9‐month‐old (c) and 13‐month‐old (d) mice. 3‐, 6‐ and 9‐month‐old groups, n = 6 each; 13‐month‐old group, n = 3. (e–h) The levels of serum total cholesterol (TC), HDL‐C, LDL‐C, total triglycerides (TG) and free fatty acids (FFA) in mice at 3‐month‐old (e, n of WT = 4, n of KAL‐TG = 5), 6‐month‐old (f, n of WT = 9, n of KAL‐TG = 8), 9‐month‐old (g, n = 8) and 13‐month‐old (h, n of WT = 5, n of KAL‐TG = 7) mice. (i) ATP levels in the gastrocnemius from mice at different ages. 3‐, 6‐ and 9‐month‐old groups, n = 6 each; 13‐month‐old group, n = 3. (j) Chronological progression of phenotypic manifestations in KAL‐TG mice. (k) qPCR results of hepatic Serpina4 (n = 5). *p < 0.05 and **p < 0.01. ns, no significant differences were observed. Figure S3: Pathological upregulation of KAL promotes aberrant de novo lipogenesis through AMPK. (a) KAL levels in the culture supernatant of adenovirus‐treated myotubes (n = 3). (b) Relative fatty acid uptake in myotubes following adenovirus treatment (n = 5). (c–d) Representative immunoblot of lipid metabolism regulators in the gastrocnemius of 6‐month‐old mice: (c) CD36, FATP4 and p‐HSL/HSL; (d) FASN, ATGL and CGI58. (e) Representative immunoblots and quantification of p‐ACC/ACC and FASN in the gastrocnemius of 13‐month‐old [file JCSM-17-e70261-s002.pdf]

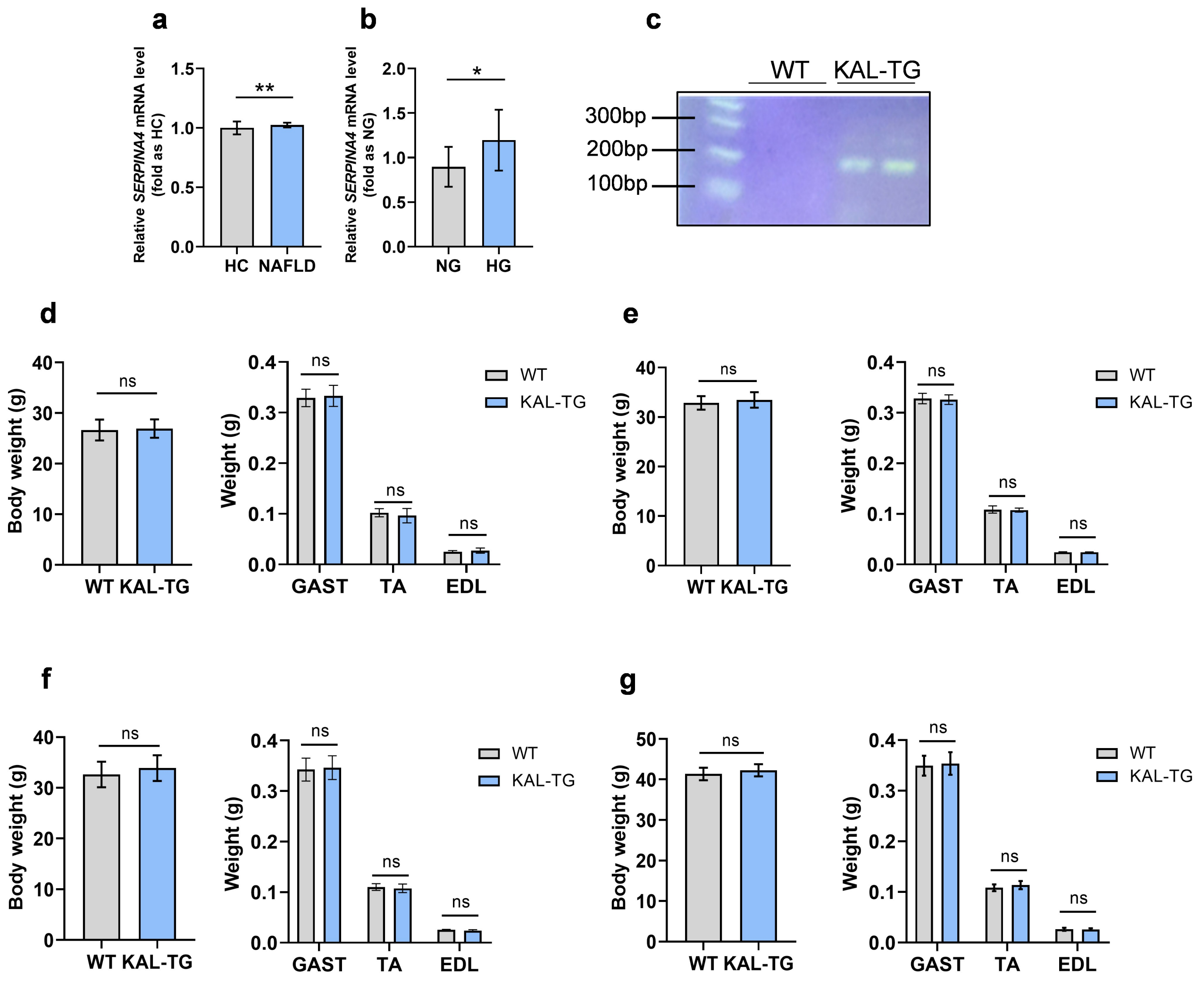

Supplementary Figure1

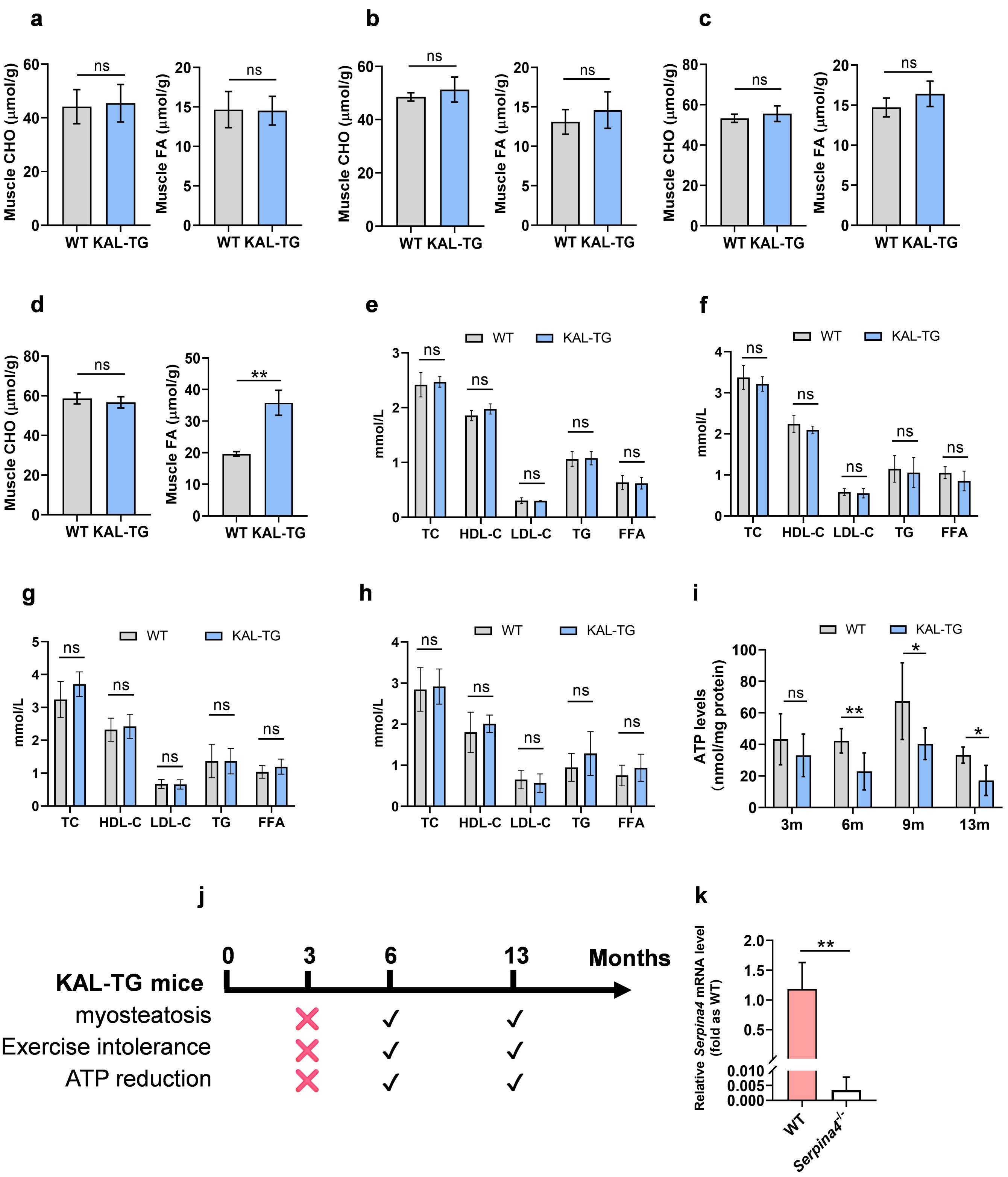

Supplementary Figure2

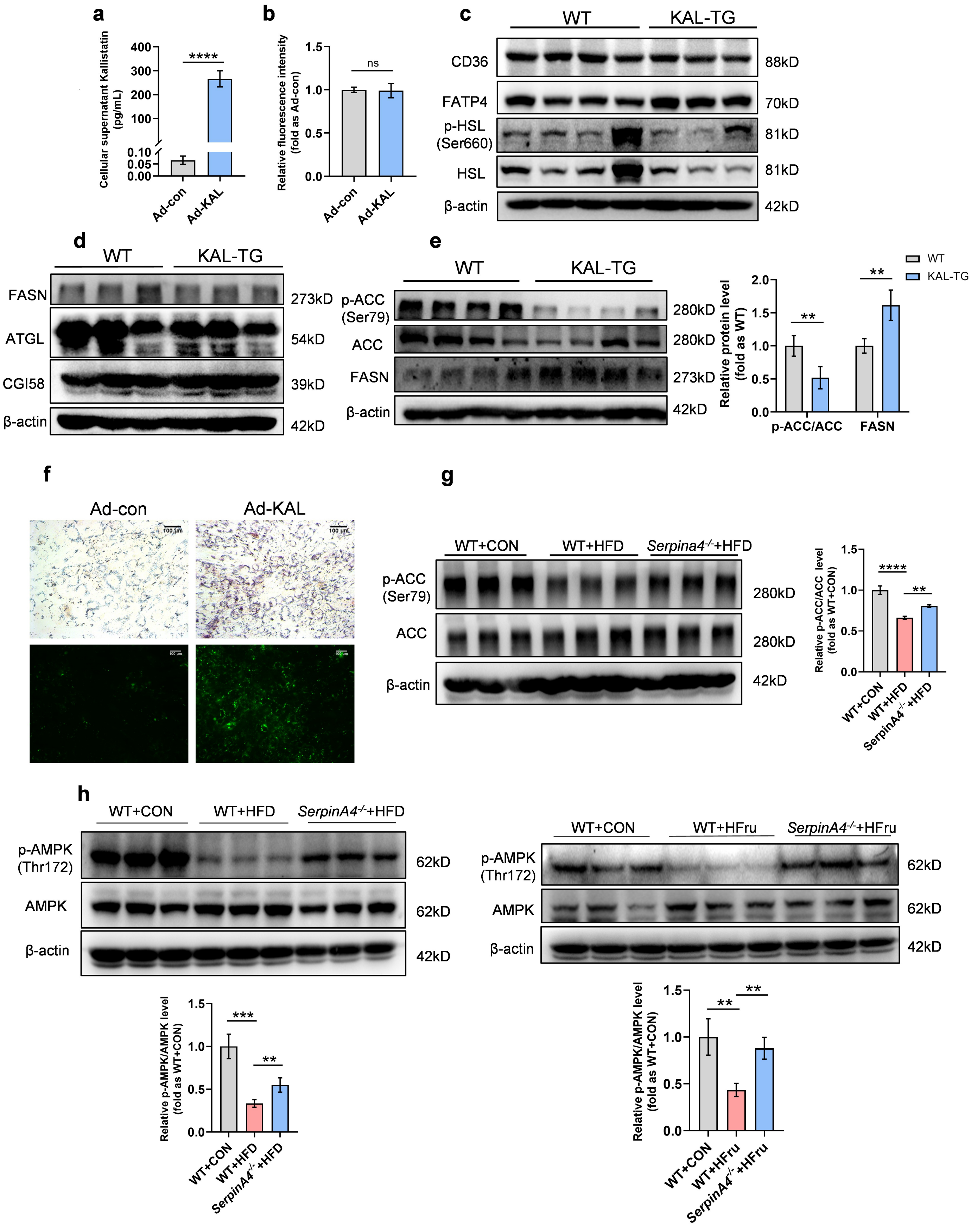

Supplementary Figure3

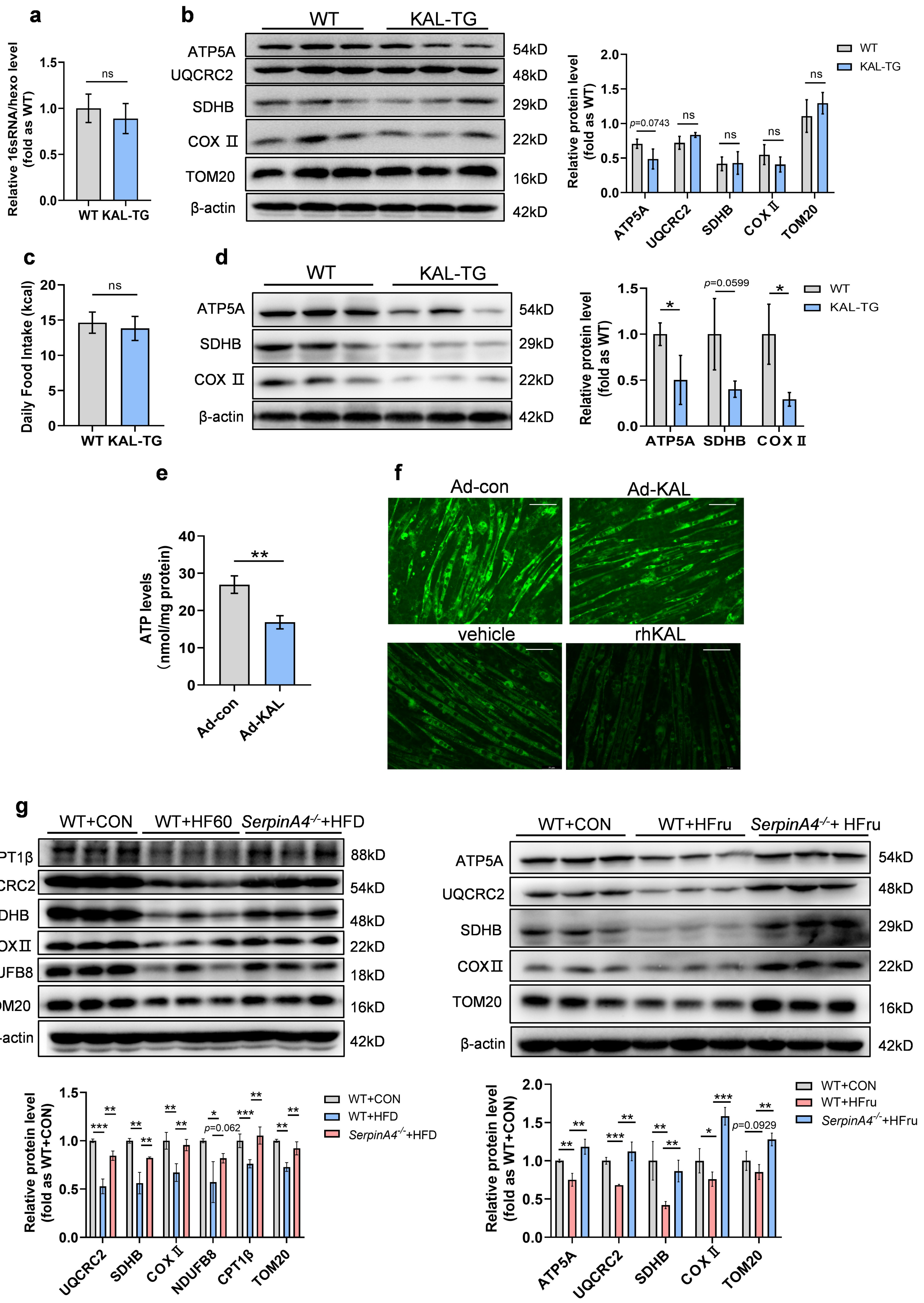

Supplementary Figure4

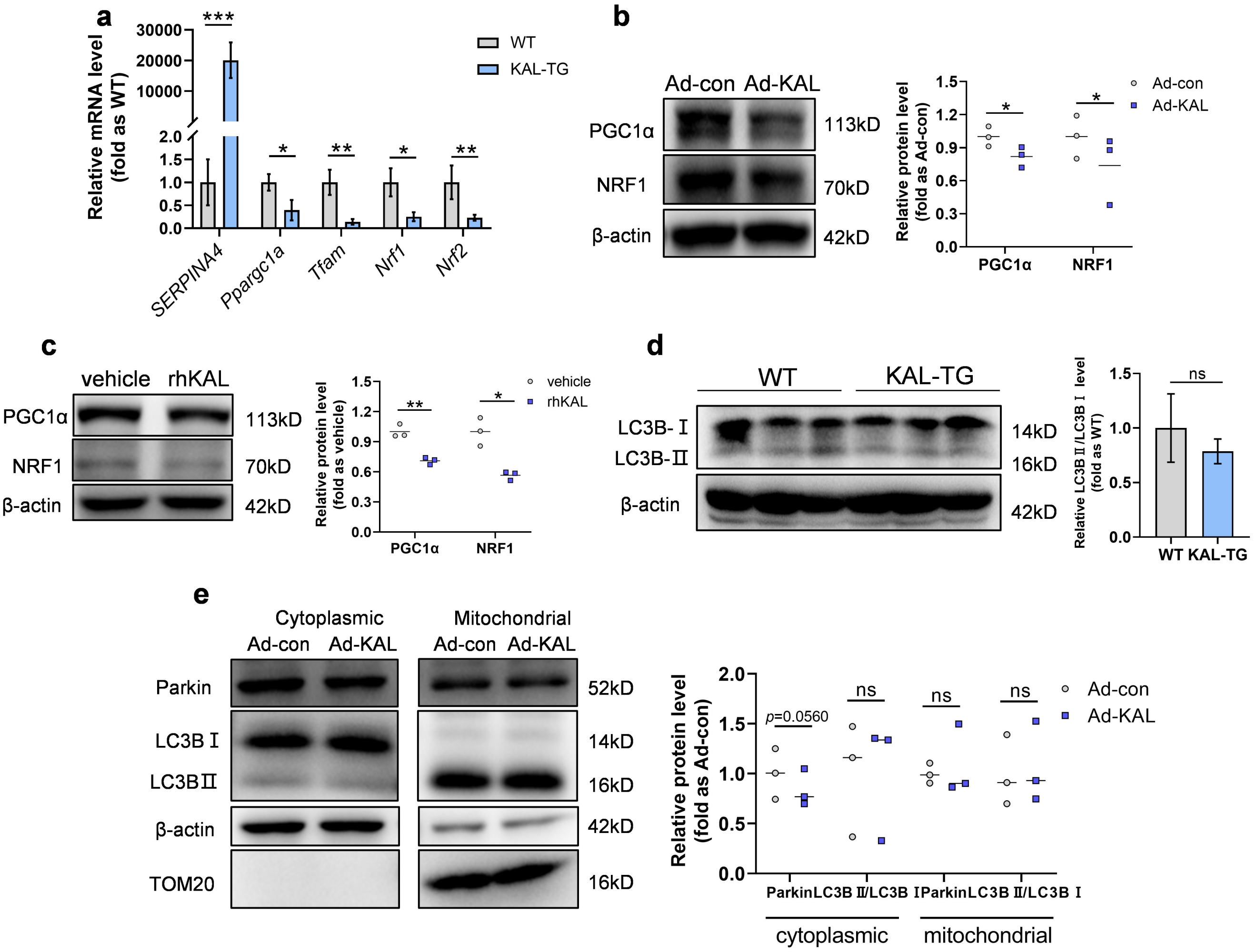

Supplementary Figure5

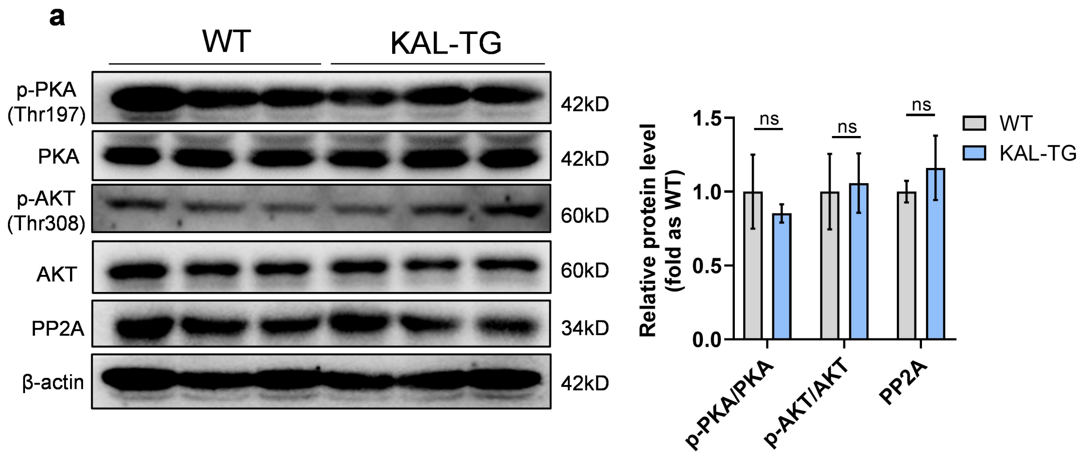

Supplementary Figure6

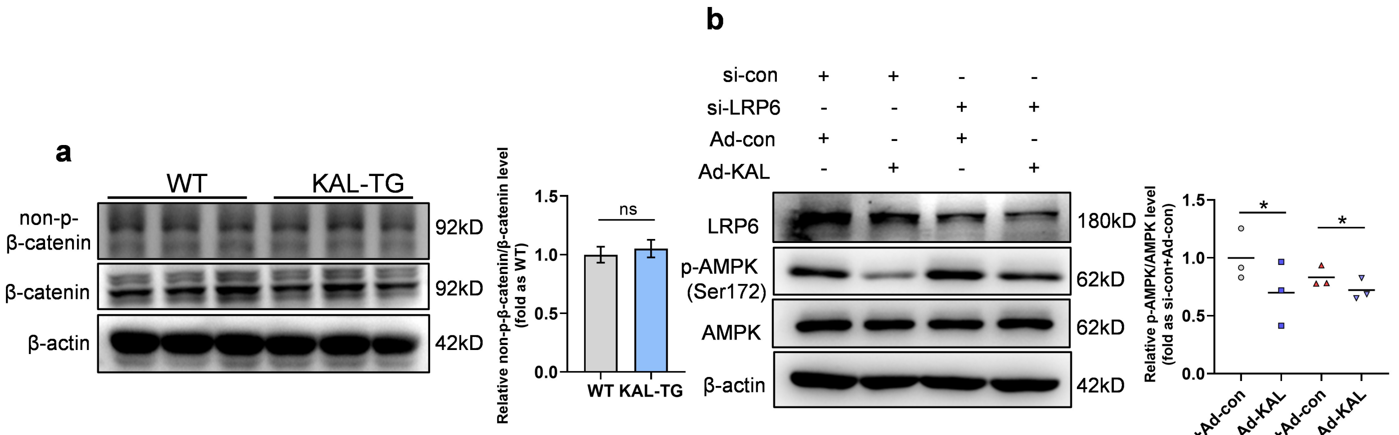

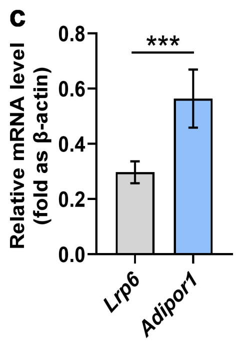

d

Web	H dock		PPA-Pred	
Receptor	Docking score	Confidence score	Kd(M)	ΔG(kcal/mol)
LRP6	-295.18	0.9480	**1.58e-5**	**-6.55**
AdipoR1	-363.22	0.9861	**1.74e-10**	**-13.31**

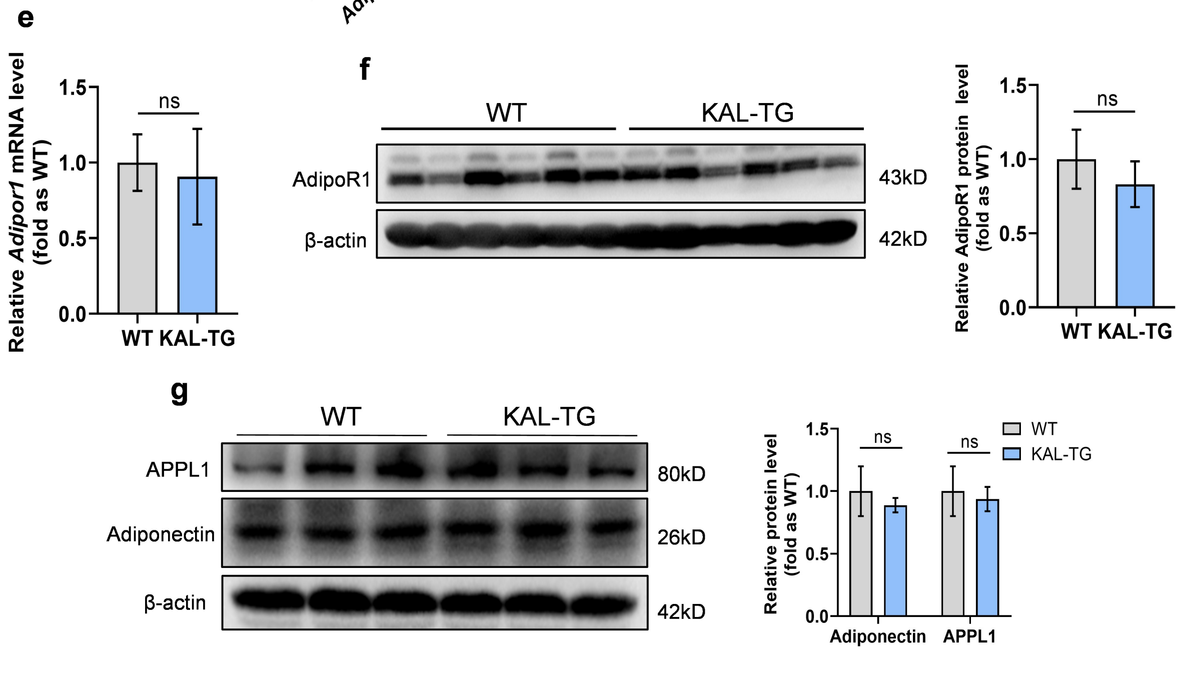

Supplementary Figure7
